# Periostin deficiency attenuates kidney fibrosis in diabetic nephropathy by improving pancreatic β-cell dysfunction and reducing kidney EMT

**DOI:** 10.1038/s41598-023-44177-5

**Published:** 2023-10-16

**Authors:** Ara Cho, Wencheng Jin, Jeonghwan Lee, Nayeon Shin, Myoung Seok Lee, Lilin Li, Seung Hee Yang, Kyong Soo Park, Chul Woo Yang, Dong Ki Kim, Yun Kyu Oh, Chun Soo Lim, Jung Pyo Lee

**Affiliations:** 1 Translational Medicine Major, Seoul National University College of Medicine, Seoul, Republic of Korea; 2 Department of Internal Medicine, Seoul National University Boramae Medical Center, 20, Boramae-ro 5-gil, Dongjak-gu, Seoul, 07061 Republic of Korea; 3 Department of Internal Medicine, Seoul National University College of Medicine, Seoul, Republic of Korea; 4 Department of Radiology, Seoul National University Boramae Medical Center, Seoul, Republic of Korea; 5 Biomedical Research Institute, Seoul National University Hospital, Seoul, Republic of Korea; 6 Kidney Research Institute, Seoul National University, Seoul, Republic of Korea; 7 Department of Internal Medicine, Seoul National University Hospital, Seoul, Republic of Korea; 8 Department of Critical Care Medicine, Yanbian University Hospital, Yanji, Jilin China; 9 Transplantation Research Center, Department of Internal Medicine, Seoul St. Mary’s Hospital, College of Medicine, The Catholic University of Korea, Seoul, Korea

**Keywords:** Diseases, Endocrinology, Medical research, Nephrology

## Abstract

Diabetic nephropathy (DN) is associated with kidney fibrosis. A previous study revealed that periostin (POSTN) contributes to kidney fibrosis. This study examined the role of POSTN in DN. The urinary concentrations of POSTN and TNC increased according to the severity of DN in human samples. Streptozotocin (STZ) was administered after unilateral nephrectomy (UNXSTZ) to induce DN in wild-type and *Postn*-null mice. Four experimental groups were generated: wild-typeham (WT Sham), wild-type UNXSTZ (WT STZ), *Postn-*null Sham (KO Sham), and *Postn*-null UNXSTZ (KO STZ). After 20 weeks, the KO STZ group had lower levels of urine albumin excretion, glomerular sclerosis, and interstitial fibrosis than those of the WT STZ group. Additionally, the KO STZ group had lower expression of fibrosis markers, including TNC. The KO STZ group showed better glucose regulation than the WT STZ model. Furthermore, the KO STZ group exhibited significantly preserved pancreatic islet integrity and insulin expression. HK-2 cells were used to observe the aggravation of fibrosis caused by POSTN under TGF-β conditions. We stimulated INS-1 cells with streptozotocin and evaluated the viability of these cells. The anti-POSTN antibody treatment of INS-1 cells with streptozotocin resulted in higher cell viability than that with treatment with streptozotocin alone. The absence of POSTN in DN contributes to renal fibrosis alleviation by improving pancreatic β-cell function. Additionally, there is an association between POSTN and TNC.

## Introduction

Diabetic nephropathy (DN) is the most common cause of chronic kidney disease (CKD)^1,2^, which is characterized by glomerulosclerosis and tubulointerstitial fibrosis, regardless of the cause^3^. At present, there is no specific treatment method for DN other than controlling glucose levels and blood pressure well before its onset.

Periostin (POSTN), a type of extracellular matrix protein, is found mainly in bones during growth. Its existence disappears after reaching adulthood^4,5^. Thereafter, it can appear in a variety of tissues in disease states. For example, the expression of POSTN has been associated with a variety of pathological conditions, including asthma^6^, heart failure^7^, myocardial infarction^8^, and metastases of various cancers^9–11^.

There has been emerging interest in the study of POSTN in the field of nephrology. Several studies have demonstrated that urinary POSTN levels are associated with tubular damage in renal fibrosis, as it is mainly expressed in tubulointerstitial areas^12^. There is growing evidence that POSTN can be effectively used as a tissue or urinary biomarker for the diagnosis of type 2 diabetes^13^, lupus nephritis^14^, and IgA nephropathy^15^.

Fibrotic tissues express high levels of POSTN^16,17^. It has also been shown that POSTN is strongly correlated with the severity of CKD^18^. A previous study reported that experimental inhibition of POSTN attenuates kidney fibrosis^19^. Additionally, POSTN promotes kidney fibrosis through the p38 MAPK pathway after acute kidney injury triggered by hypoxia or ischemia‒reperfusion injury^20^. Based on a recent study, old mice are more likely to develop kidney dysfunction than normal adults, which increases the likelihood of POSTN expression in old mice. Interestingly, old mice in which POSTN was deleted showed renal function similar to that of normal adults^21^.

However, there has been little research on the role of POSTN in DN. This study investigated the role of POSTN in the kidney and pancreas in DN models induced by streptozotocin (STZ).

## Results

### Association between kidney function and urinary POSTN and TNC levels in patients with DN

**Figure 1:**
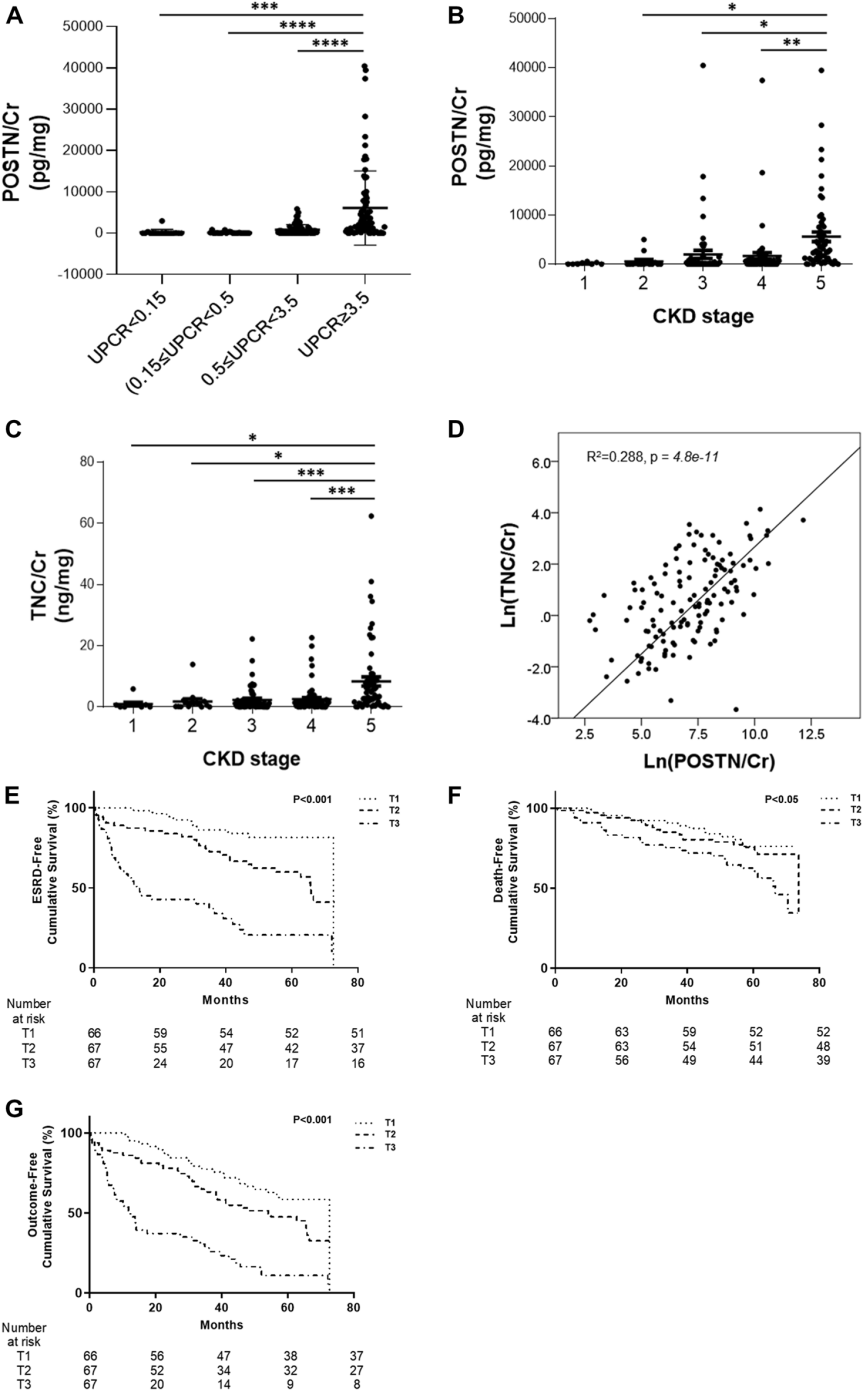
Periostin and tenascin C levels measured in urine in patients with diabetic nephropathy. (**A**) The concentration of POSTN in urine based on the UPCR value (N = 207). (**B**) POSTN urine concentration by CKD stage in DN patients (N = 199). Data are the mean ± SD. *p < 0.05, ***p < 0.001 (one-way ANOVA). (**C**) TNC urine concentration by CKD stage in DN patients (N = 207). Data are the mean ± SD. *p < 0.05, ***p < 0.001 (one-way ANOVA). (**D**) Correlation of POSTN/Cr and TNC/Cr. (**E**) Kaplan‒Meier patient survival curves for ESRD. (**F**) Kaplan‒Meier patient survival curves for death. (**G**) Kaplan‒Meier patient survival curves for composite.

Measurement of POSTN and TNC concentrations in the urine of 200 DN patients was performed using ELISA. This study classified patients into a DN group according to the UPCR and eGFR. First, the patients were divided into four groups: UPCR < 0.15 g/g; mild proteinuria (0.15 ≤ UPCR < 0.5 g/g); moderate proteinuria (0.5 ≤ UPCR < 3.5 g/g); and massive proteinuria (UPCR ≥ 3.5 g/g). Based on the UPCR level, the POSTN concentration in urine increased proportionally (p < 0.0001) (Fig. 1A). Next, the patients were divided into five groups according to the CKD stage, and the POSTN concentration increased with an increase in the CKD stage (p = 0.0012) (Fig. 1B). The TNC concentration increased as the CKD stage increased (p < 0.0001) (Fig. 1C). The TNC concentration increased as the POSTN concentration increased. There was a positive correlation between log(POSTN/Cr) and log(TNC/Cr). (*R*^2^ = *0.288, p* < *0.001*) (Fig. 1D). Based on a median follow-up of 49.8 ± 19.6 months, 101 (46.1%) patients were diagnosed with ESRD, and 65 (29.7%) patients died. Patients were divided into three groups based on their POSTN/Cr concentration (T1: POSTN/Cr < 76.8; T2: 76.8 ≤ POSTN/Cr < 1458.5; and T3: POSTN/Cr ≥ 1458.5). A Kaplan‒Meier survival analysis was conducted to investigate the association between the urinary soluble POSTN concentration and ESRD or mortality. Patients with a high urine POSTN/Cr level had an increased risk of ESRD (Fig. 1E) and death (Fig. 1F), as well as a composite outcome (Fig. 1G), when compared with patients with a low urinary POSTN/Cr level. The risk of ESRD and the composite score increased for patients with high POSTN/Cr concentrations (*p* < *0.001*). The risk of death was elevated for patients with high POSTN/Cr concentrations (*p* < *0.05*).

### Effect of POSTN deficiency on reducing proteinuria and elasticity in the DN animal model

**Figure 2:**
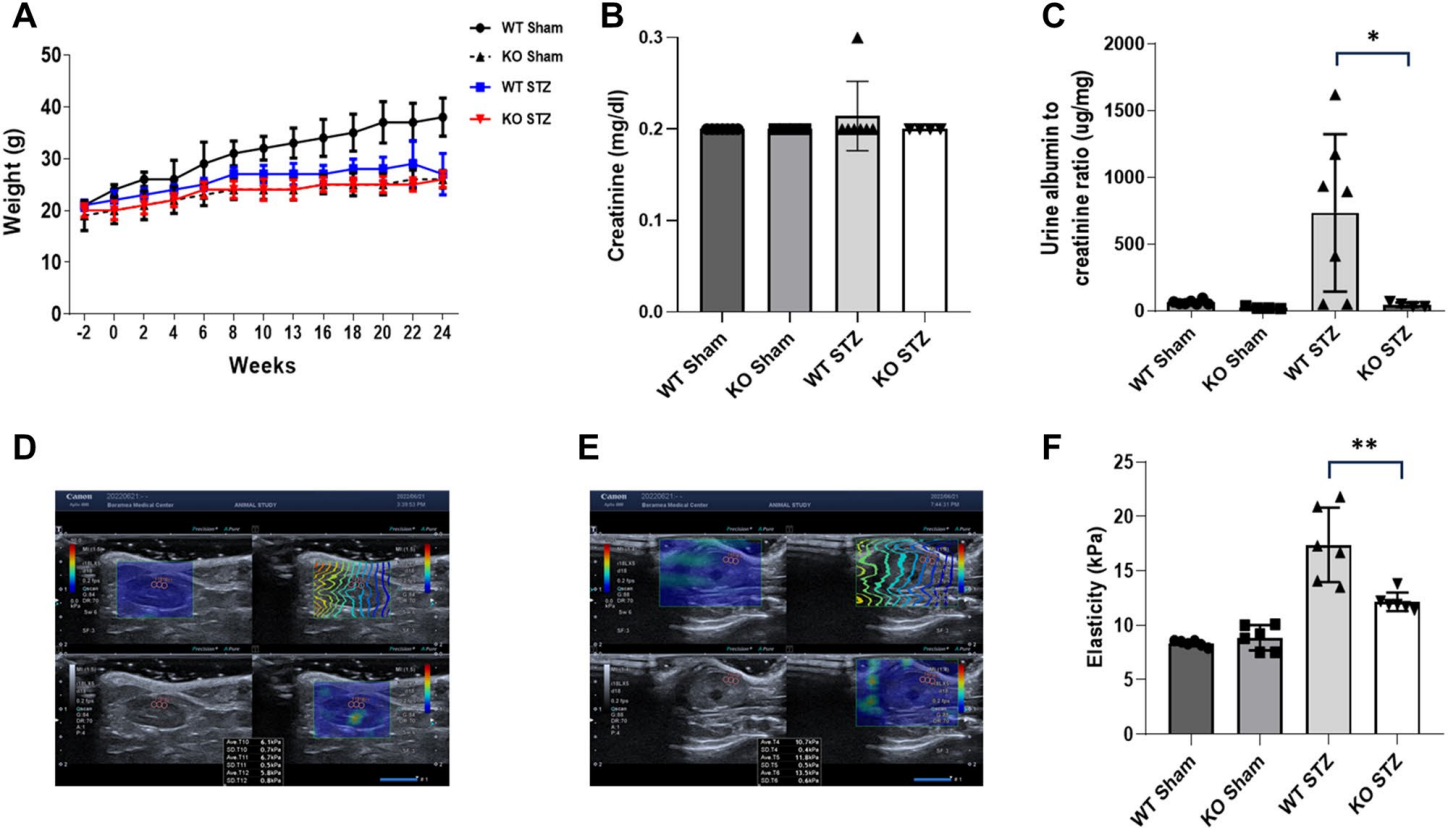
Functional data of the mouse groups. (**A**) A graph of the weight changes by group for 24 weeks (WT Sham, WT STZ: N = 7/group; KO Sham, KO STZ: N = 4/group). (**B**) A comparison of creatinine measurements among groups of mice. (**C**) Comparative graph of the albumin/creatinine ratios among groups (WT Sham, WT STZ: N = 7/group; KO Sham, KO STZ: N = 4/group). Data are the mean ± SD. *p < 0.05 (Mann‒Whitney test). (**D**) Measurement of the shear-wave elastography (SWE) of WT Sham kidneys. (**E**) Shear-wave elastography (SWE) measurements of KO STZ kidneys. (**F**) A graph of elasticity (kPa) by group based on elastography (N = 3/group). Data are the mean ± SD. **p < 0.01 (Mann‒Whitney test).

In the WT mice, the body weight of the Sham group gradually increased for 24 weeks; however, the body weight of UNXSTZ did not increase (Fig. 2A). At 24 weeks, the difference in body weight was significant between the two groups. On the other hand, in POSTN KO mice, body weight in both the Sham and UNXSTZ groups did not increase; therefore, no significant difference was seen between the two groups. Creatinine levels were not different among the groups (Fig. 2B). The albumin-to-creatinine ratio in urine was also elevated in WT STZ mice, whereas for the KO STZ group, there was no significant difference between them and KO Sham mice (Fig. 2C).

Shear-wave elastography (SWE) measurements were made of Sham kidneys (Fig. 2D) and KO STZ kidneys (Fig. 2E). In each capture, the upper right image is a stiffness map (kPa), the upper left image is a propagation map that shows the propagation of shear waves using contour lines, the lower left image is a variation (confidence) map that represents low variation (high confidence) in the stiffness value as a blue color, and the lower right image is a grayscale two-dimensional ultrasound image. According to elastography measurements of intrarenal elasticity, the WT STZ group demonstrated statistically significant increases in elasticity, in contrast to the KO STZ group. Comparing the KO STZ group with the WT Sham and KO Sham groups showed a slight increase in elasticity, but similar elasticity was observed (Fig. 2F).

### Antifibrotic effect of POSTN deficiency in the DN animal model

**Figure 3:**
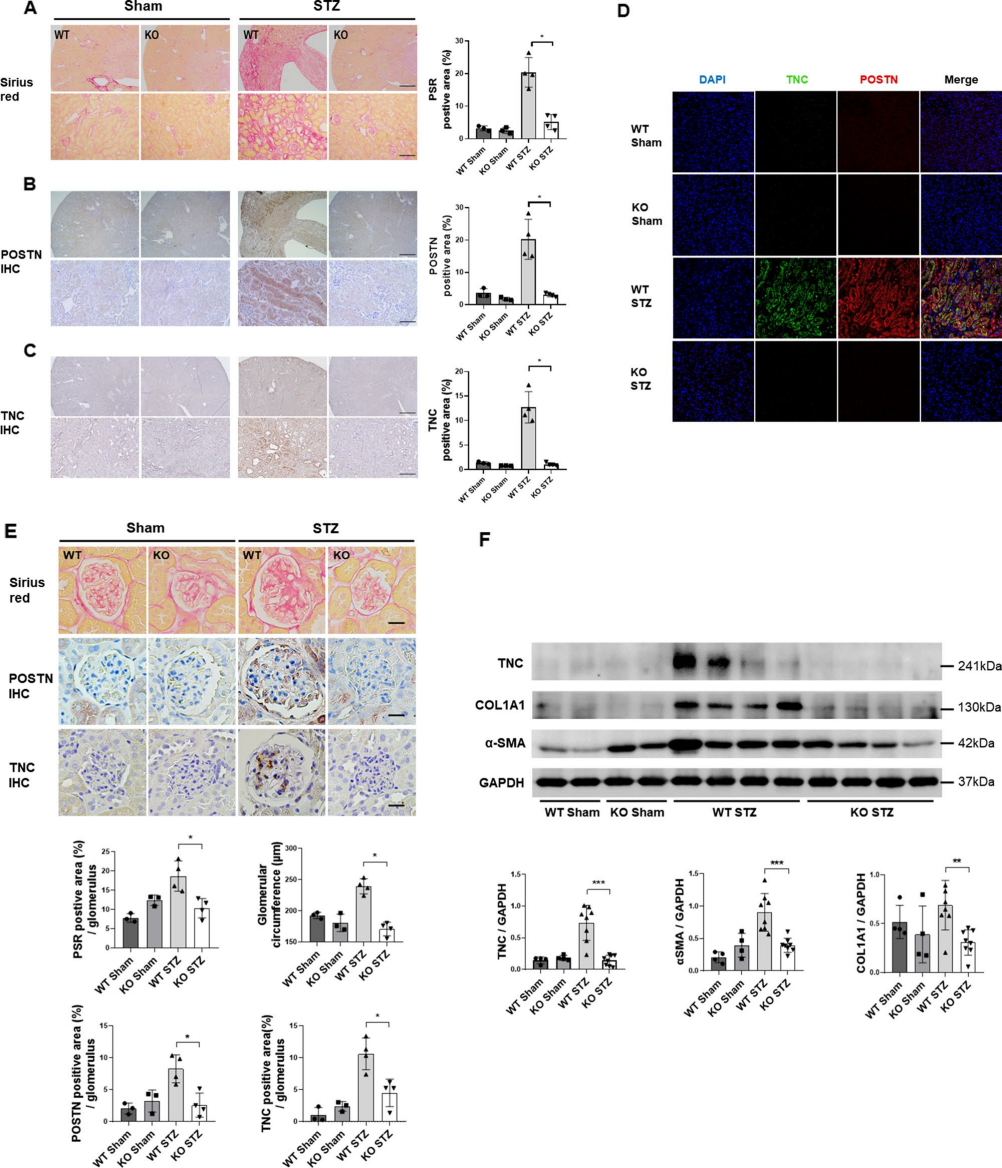
Analysis of data related to fibrosis among the groups of mice. (**A**) The degree of fibrosis progression was compared between groups based on Sirius red staining (WT Sham, KO Sham: N = 3/group; WT STZ, KO STZ: N = 4/group). Data are the mean ± SD. *p < 0.05 (Mann‒Whitney test). (**B**) Results of POSTN IHC staining by group (WT Sham, KO Sham: N = 3/group; WT STZ, KO STZ: N = 4/group). Data are the mean ± SD. *p < 0.05 (Mann‒Whitney test). Bar = 500 μm. Bar = 100 μm. (**C**) Results of TNC IHC staining by group (WT Sham, KO Sham: N = 3/group; WT STZ, KO STZ: N = 4/group). Data are the mean ± SD. *p < 0.05 (Mann‒Whitney test). Bar = 500 μm. Bar = 100 μm. (**D**) The result of the concurrent confirmation of POSTN and TNC in the tubule of the kidney tissue is presented by immunofluorescence staining. (**E**) The degree of intraglomerular fibrosis progression between groups was investigated using Sirius red staining and IHC staining. The glomerular circumference of each group was measured and analyzed (WT Sham, KO Sham: N = 3/group; WT STZ, KO STZ: N = 4/group). Data are the mean ± SD. *p < 0.05 (Mann‒Whitney test). Bar = 100 μm. (**F**) A comparison of fibrosis marker expression levels by group as assessed by western blotting (WT Sham, KO Sham: N = 4/group; WT STZ, KO STZ: N = 8/group). Data are the mean ± SD. **p < 0.01, ***p < 0.001 (Mann‒Whitney test). Original blots/gels are presented in Supplementary Figs. 1b.

Sirius red staining revealed that a significant amount of fibrosis had developed in the WT STZ group compared to the KO STZ group (Fig. 3A**).** Both POSTN and TNC immunohistochemistry (IHC) showed that the expression levels of POSTN and TNC were also significantly higher in the WT STZ group. (Fig. 3B,C). The expression levels of POSTN and TNC were found to be high in the tubules of WT STZ mice by immunofluorescence staining (Fig. 3D). Several measures were taken to assess the degree of fibrosis within the glomerulus. The Sirius red staining results confirmed that the degree of fibrosis progression in the glomerulus of the WT STZ group was higher than that of the other groups. By examining the degree of intraglomerular TNC staining with IHC, it was determined that the WT STZ group possessed significantly higher levels of intraglomerular TNC accumulation than the other groups. Based on the IHC evaluation of the staining degree of POSTN in the glomeruli, the WT STZ group displayed the strongest staining degree (Fig. 3E). According to the western blot results, Fibronectin, COL1A1, and *α-SMA*, representative fibrosis markers, were increased in the WT STZ group compared to the WT Sham group. The expression level of TNC, which is thought to be related to POSTN, was also higher in the WT STZ group (Fig. 3F).

### Effect of POSTN deficiency on the TGF-β pathway in a mouse model of kidney fibrosis

**Figure 4:**
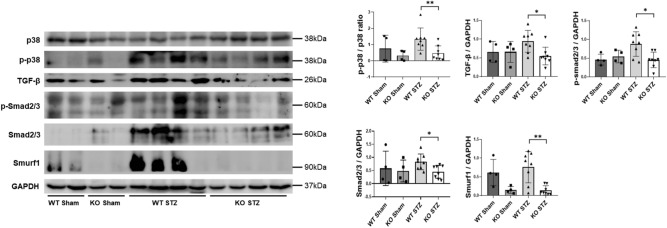
The effect of periostin deficiency on the TGF-β pathway in a diabetic nephropathy model. The expression levels of TGF-β signaling-related markers differed among the study groups, as shown by the western blot results (WT Sham, KO Sham: N = 4/group; WT STZ, KO STZ: N = 8/group). Data are the mean ± SD. *p < 0.05, **p < 0.01 (Mann‒Whitney test). Original blots/gels are presented in Supplementary Fig. 2b, 3b.

In contrast to the other groups, the WT STZ group showed significant increases in the expression levels of signaling proteins related to the TGF-β pathway. According to the study, the expression level of TGF-β increased, resulting in the upregulation of sub proteins. Moreover, in the KO STZ group, the expression level of TGF-β was similar to that of the Sham group, and the expression levels of the sub proteins were also maintained at a similar level. This study found that the quantitative differences in these TGF-β-related signals explained the differences in the fibrosis severity by group (Fig. 4).

### Effect of POSTN on an in vitro kidney model

**Figure 5:**
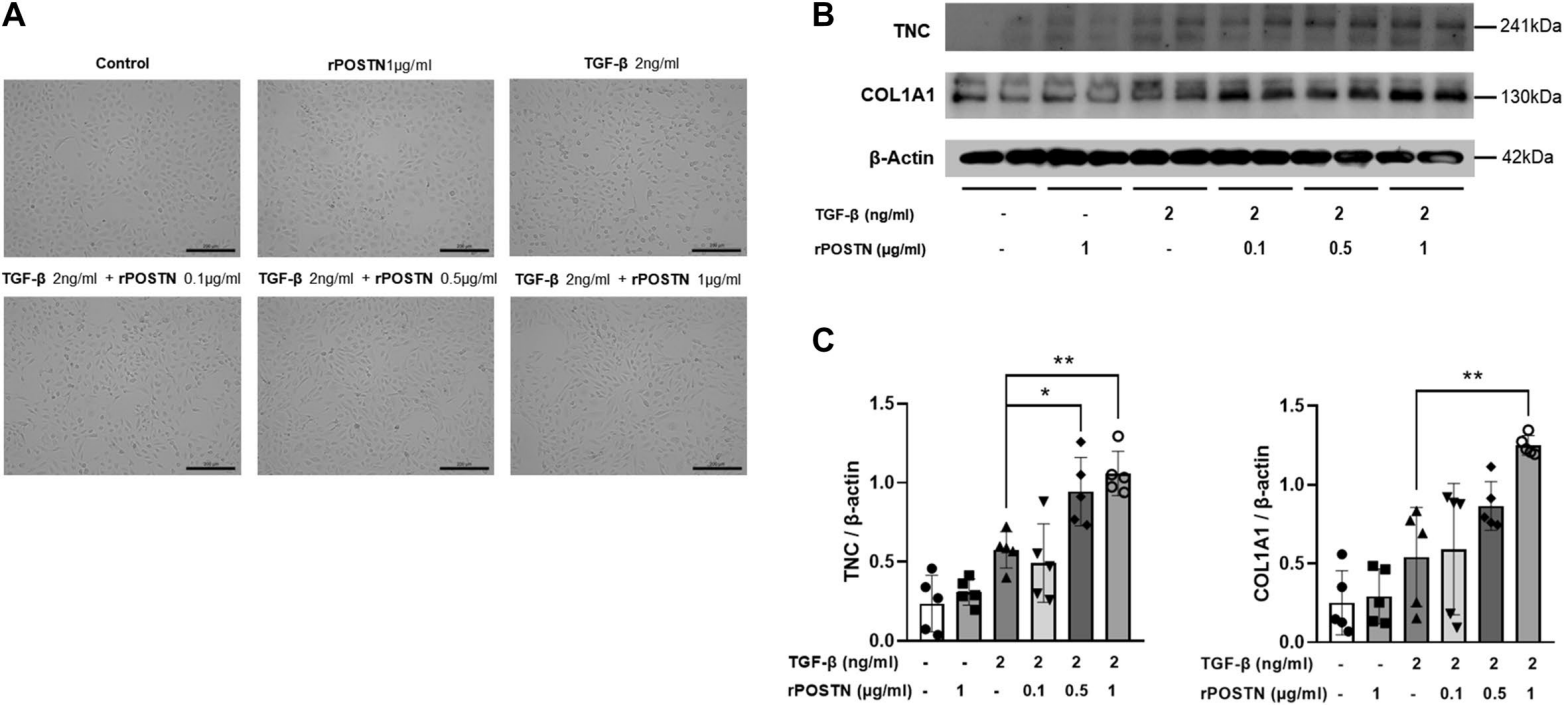
TGF-β and periostin-induced fibrosis in HK-2 cells. (**A**) Morphological changes in HK-2 cells after treatment with TGF-β and rPOSTN. Bar = 200 μm. (**B**,**C**) Western blot results presenting a change in the expression of TNC and COL1A1 when HK-2 cells were treated with TGF-β and POSTN simultaneously or separately (N = 5/group). Data are the mean ± SD. *p < 0.05, **p < 0.01 (one-way ANOVA). Original blots/gels are presented in Supplementary Fig. 4b.

To investigate whether rPOSTN exacerbates TGF-β-induced fibrosis, HK-2 cells were treated with TGF-β and rPOSTN simultaneously. The morphology of the cells was observed in response to each treatment condition (Fig. 5A). Fibrosis was not observed with rPOSTN treatment alone; however, fibrosis was significantly aggravated when cells were treated with TGF-β and rPOSTN simultaneously compared with TGF-β alone (Fig. 5B,C). In addition, fibrosis worsened with the dose of rPOSTN when the dose of TGF-β was fixed (Fig. 5B,C).

### Improving pancreatic β-cell dysfunction by POSTN deficiency

**Figure 6:**
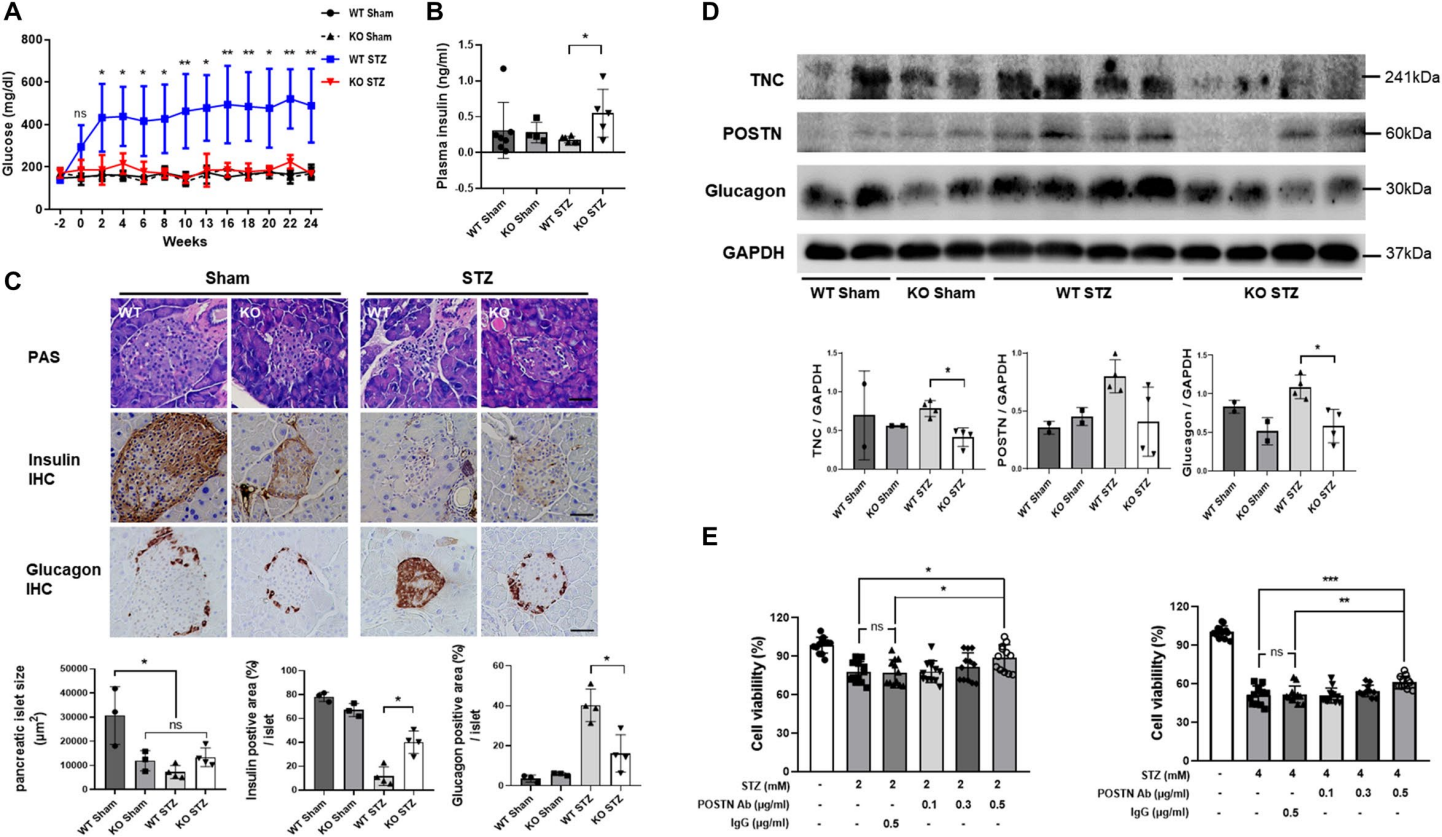
The role of POSTN in pancreatic function. (**A**) Changes in blood glucose levels by group observed during the 24-week study (WT Sham, WT STZ: N = 7/group; KO Sham, KO STZ: N = 4/group). Data are the mean ± SD. *p < 0.05, **p < 0.01 (Mann‒Whitney test). (**B**) A comparison of the blood insulin levels between groups (WT Sham, WT STZ: N = 6/group; KO Sham, KO STZ: N = 4/group). Data are the mean ± SD. *p < 0.05 (Mann‒Whitney test). (**C**) Morphological changes between groups of pancreatic islets as evidenced by PAS staining (WT Sham, KO Sham: N = 3/group; WT STZ, KO STZ: N = 4/group). The IHC staining results indicate differences between groups in insulin and glucagon expression. Data are the mean ± SD. *p < 0.05 (Mann‒Whitney test). Bar = 50 μm. (**D**) A comparison of POSTN, TNC, insulin, and glucagon levels in pancreatic tissue by group (WT Sham, KO Sham: N = 2/group; WT STZ, KO STZ: N = 4/group). Data are the mean ± SD. *p < 0.05 (Mann‒Whitney test). Original blots/gels are presented in Supplementary Fig. 5b. (**E**) An analysis of INS-1 cell viability when treated with STZ and neutralizing POSTN antibodies (N = 12/group). Data are the mean ± SD. *p < 0.05, **p < 0.01, ***p < 0.001 (one-way ANOVA).

The blood glucose concentration increased in WT STZ 2 weeks after STZ injection, whereas it remained within the normal range in KO STZ. The difference in blood glucose levels between the two groups 2 weeks after STZ injections was significant (Fig. 6A). For the blood insulin concentration, the WT STZ group had a significantly lower insulin concentration than the other groups, while the KO STZ group maintained a high blood insulin concentration, indicating the reason for the difference in blood insulin concentration (Fig. 6B).

When the size of islets in the pancreas was compared between the WT STZ group and the other groups, it was found that the size of the islets was significantly reduced in the WT STZ group. The KO STZ group, however, showed islet sizes similar to those observed in the KO Sham group. Furthermore, it was evident that the integrity of the islet had been preserved in the KO STZ group; however, this was not the case in the WT STZ group. In addition, when the insulin expression level in the islets was compared, the expression level of insulin sharply decreased in the WT STZ group, while the expression level of insulin in the KO STZ group was similar to that of the KO Sham group. A high level of glucagon expression was also observed in the WT STZ group, while in the KO STZ group, it was maintained at a level similar to that of the KO Sham group (Fig. 6C). Compared to the other groups, the WT STZ group showed the highest expression levels of TNC and POSTN in the pancreas, while the KO STZ group demonstrated similar expression levels to the Sham group. In the WT STZ group, the level of glucagon expression was the highest, and in the KO STZ group, it was similar to the level of expression in the Sham group (Fig. 6D).

The apoptosis rate increased in proportion to the STZ concentration when STZ was administered to insulin-producing INS-1 cells. In the STZ-treated state, INS-1 cells became more viable as more anti-POSTN IgG treatment was applied. INS-1 cells treated with isotype IgG in addition to STZ had no statistically significant difference in viability from cells treated with STZ alone. As a result of anti-POSTN IgG treatment, cell viability increased, but it was not just an antibody effect (Fig. 6E).

## Discussion

The purpose of this study was to examine the prognostic significance of POSTN levels in patients with DN. There was a significant association between high POSTN levels and poor kidney function. The role of POSTN in DN was also examined through UNXSTZ (in vivo model) and experiments using HK-2 and INS-1 cells (in vitro models). As a result of the experiment, it was possible to prevent kidney fibrosis and lower blood sugar levels in individuals with disease by blocking POSTN. In this study, the patients were divided into four groups on the basis of their UPCR values: UPCR < 0.15 g/g; mild proteinuria (0.15 ≤ UPCR < 0.5 g/g); moderate proteinuria (0.5 ≤ UPCR < 3.5 g/g); and massive proteinuria (UPCR ≥ 3.5 g/g)^22^. Additionally, the same patients as above were divided into five groups according to CKD stage. This study found that the urine POSTN concentration increased as the UPCR level and stage of CKD increased in the DN patient group. According to the results of the above experiments, urinary POSTN concentration increased with the deterioration of renal function in patients with DN. These findings were similar to those of a previous study conducted in nondiabetic CKD and IgA nephropathy^15^. In CKD, POSTN plays an important role in the development of kidney fibrosis^20^. This study is consistent with previous studies. To determine the roles of POSTN in kidney fibrosis in DN, this study was conducted.

As a matricellular protein (MCP), TNC was reported to play a role as a biomarker for diabetes^23^. It is also known as a factor that plays an important role in the progression of CKD, similar to POSTN^24,25^. Furthermore, TNC and POSTN exchange positive feedback with each other to regulate each other’s expression levels^26^. It has been shown that TNC concentrations are associated with kidney impairment in patients with DN. In mouse kidneys with DN, as well as in the pancreas, there is a quantitative correlation between TNC and POSTN. Additionally, in cell experiments, TNC expression levels increased in proportion to the POSTN treatment concentrations in the presence of TGF-β. In future studies, it will be important to go beyond the quantitative correlation between the two substances to unveil the correlation of specific molecular mechanisms of each substance in the kidneys and pancreas.

According to our results, DN-induced fibrosis was less common in the KO STZ groups than in the WT STZ groups. Fibrosis associated with DN can be explained using the TGF-β pathway^27^. This study found a difference in the expression level of TGF-β-related signals according to the presence or absence of POSTN. Previous studies have shown that POSTN and TGF-β are closely related to CKD, which means that when TGF-β increases the expression of POSTN, the increased POSTN induces inflammatory cytokines, and consequently, TGF-β levels increase^20^. Thus, POSTN and TGF-β are interconnected in a vicious cycle. In CKD caused by DM, the TGF-β-related mechanism may play a major role similar to that in nondiabetic CKD.

The p38 MAPK enzyme regulates several cellular functions in response to extracellular stimulation, including gene expression, mitosis, differentiation, migration, survival, and apoptosis. A previous study investigated the association between the p38 MAPK pathway and POSTN in the progression of AKI to CKD^20^. This study investigated the association between POSTN and p38 MAPK in DN using western blotting. In the WT STZ group, the expression level of pp38 MAPK was higher, whereas the expression level of ppMAPK38 was significantly lower in the KO STZ group. Consequently, there were also differences in the degree of fibrosis between the two groups.

Prior studies have demonstrated that POSTN induces fibrosis in tubular epithelial cells, and this has been shown to be a direct cause of kidney fibrosis^20^. In this study, a proximal tubular cell line, HK2, was used for cell experiments. In the presence of TGF-β, POSTN directly promoted fibrosis of tubule cells. Based on these results, POSTN may exacerbate DN by influencing renal EMT.

In the present study, it was also found that *Postn*-null mice did not have hyperglycemia because they exhibited preserved insulin secretory function even after STZ injections compared with WT mice. Additionally, when INS-1 cells and pancreatic islet β-cells were treated with a neutralizing POSTN antibody under STZ-treated conditions, their viability was improved. According to the results of the experiment, POSTN results in a reduction in the secretion of insulin. To our knowledge, our work is the first to demonstrate that POSTN directly plays a role in the deterioration of pancreatic functions, although the exact mechanism by which POSTN affects pancreatic cell viability remains unidentified at the molecular level.

One previous study showed contradictory results wherein *Postn*-null mice exhibited impaired mesenchymal formation of the pancreas and reduced regeneration after partial pancreatectomy^28^. In that study, the *Postn*-null mouse group also showed increased blood glucose, decreased insulin secretion, and fewer beta cells than the WT mice^28^. Another study also showed that *Postn-*null mice in the acute pancreatitis model did not recover their exocrine pancreatic function well^29^. POSTN plays a crucial role in exocrine regeneration after severe acute pancreatitis^29^. Both papers examined the role of POSTN in pancreatic structure and function, and their results contradicted our findings. Although the reason for the increased level of glucagon in the WT STZ group has yet to be identified, it remains unknown what might be responsible for it. Further research is necessary to clarify whether POSTN directly affects alpha cells and increases their concentration of glucagon, whether it is a compensatory process due to the decrease in insulin, or whether it is related to both mechanisms.

Collectively, POSTN may have a significant effect on controlling glycemic levels in patients with type 1 diabetes, as well as preventing kidney damage caused by POSTN. If an animal model study using anti-POSTN was conducted, the exact effect of POSTN on DN could be determined. As the model takes 24 weeks to complete, conducting another experiment as a part of this study would be difficult. If the experiment described above is conducted in the future, the findings in this paper will be more concrete, and there will be greater possibilities for clinical applications.

## Materials and methods

### Measurement of urine tenascin C (TNC) and POSTN levels in patients with DN

To evaluate an association between kidney function and urinary POSTN and/or TNC, the clinical data of 200 individuals with DN were collected, including age, sex, serum creatinine, estimated glomerular filtration rate (eGFR) and urine protein-to-creatinine ratio (UPCR). eGFR was calculated using the CKD-EPI creatinine equation. Over the period of January 2009 to December 2016, 218 patients with clinically diagnosed DN were enrolled in this study. Among the 218 patients, 20 had biopsy-proven disease. Those younger than 18 or planning to undergo a kidney transplant were excluded from the study. We collected demographic and clinical data from electronic medical records, such as age, sex, body mass index (BMI), systolic and diastolic blood pressure, and comorbidities (e.g. hypertension, diabetes)^30^. A POSTN ELISA kit (27,751, IBL) and TNC ELISA kit (DY3548B, R&D system) were used to measure the urinary POSTN and TNC levels. All measurements were performed in duplicate in a blinded manner. Informed consent was obtained from the study participants before enrollment. The present study was conducted with the approval of the Research Ethics Committee of the Seoul National University Boramae Medical Center (IRB no. 06-2011-50). All procedures were performed in accordance with the ethical standards of the institutional and/or national research committee and with the 1964 Declaration of Helsinki and its later amendments or comparable ethical standards.

### Experimental animals with diabetic nephropathy

C57BL/6 male mice (20–22 g, aged 7 weeks) were purchased from Koatech (Kyeonggi-do, Korea) and acclimated to the laboratory conditions for one week. A specific pathogen-free animal facility was used for raising both male WT mice and male *Postn*-null mice (C57BL/6; 129-Postntm1Jmol/J; the Jackson Laboratory, Bar Harbor, ME, USA) at 22 °C, 60–70% humidity, 5 mmH2O air pressure, 150–300 lx illumination, and a noise level of 60 dB or less, and ventilation was performed 10–20 times per hour.

**Figure 7:**
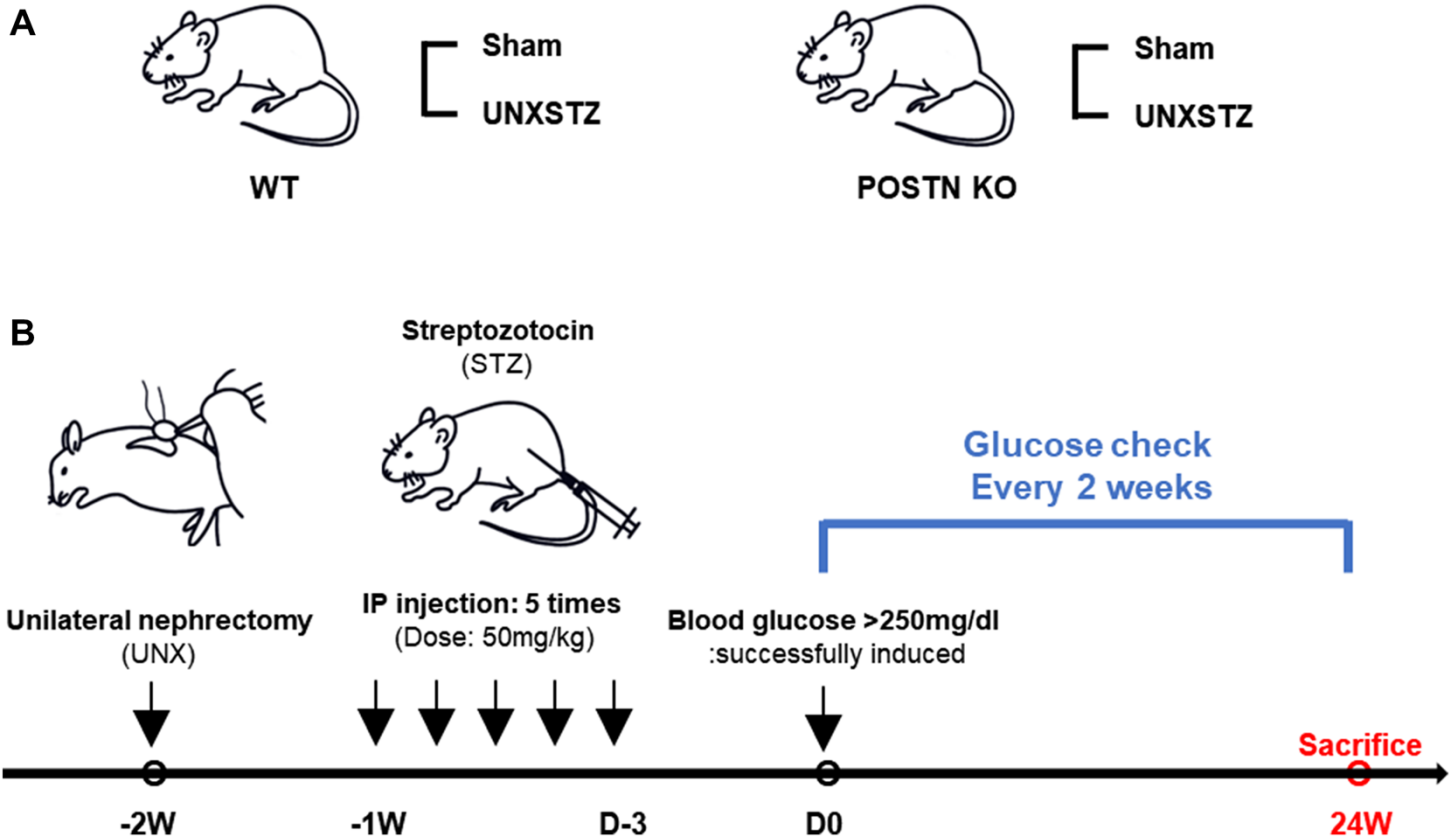
A schematic overview of the animal model. The diabetic nephropathy in vivo model was induced by streptozotocin (STZ) administration after unilateral nephrectomy (UNX). One week after UNX, the mice were injected with 50 mg/kg STZ intraperitoneally for 5 consecutive days. Tail vein blood glucose levels were measured to confirm diabetes (fasting blood glucose > 300 mg/dL). The mice were sacrificed at 24 weeks after induction of diabetes.

As a DN model, this study used the unilateral nephrectomy–streptozotocin (UNXSTZ) model, which is induced by STZ administration after unilateral nephrectomy (Fig. 7)^31^. Unilateral nephrectomy was performed on the experimental mice. One week after UNX, the mice were injected with 50 mg/kg STZ (Sigma‒Aldrich, St. Louis, MO, USA) intraperitoneally for 5 consecutive days^32^. Tail vein blood glucose levels were measured to confirm diabetes (fasting blood glucose > 300 mg/dL). The mice were sacrificed at 24 weeks after induction of diabetes^33^. A spot urine sample was collected and measured for creatinine and albumin before the mice were sacrificed. The animals were randomly divided into four experimental groups: wild-type Sham (WT Sham), wild-type UNXSTZ (WT STZ), *Postn*-null mice Sham (KO Sham), and *Postn*-null mice UNXSTZ (KO STZ).

Both the WT Sham and KO Sham groups were subjected to the same conditions as the UNXSTZ groups except for the diabetes-inducing surgical procedure. PBS was injected into both Sham groups at the same time as STZ was injected into the UNXSTZ groups. There was no difference in the frequency of changing cages or measuring blood glucose levels between the Sham groups and the STZ groups. The environment was designed so that all groups would receive the same amount of stress.

In accordance with the National Research Council’s Guidelines for the Care and Use of Laboratory Animals, the animal experiments were conducted in the animal laboratory of the Seoul National University Boramae Medical Center under pathogen-free conditions after approval from the Institutional Animal Care and Use Committee (No. 2020-0026). For animal experiments, all methods are reported according to the ARRIVE guidelines (https://arriveguidelines.org).

### Measurement of kidney function by UACR

A spot urine sample was obtained before sacrificing mice that had been maintained in the diabetic model for 24 weeks. A minimum of 100 mL of spot urine was collected. By checking the UACR level, it was confirmed that diabetic nephropathy had occurred^33^. Spot urine was used to measure albumin and creatinine using Albuwell M (Ethos Bioscience) and The Creatinine Companion (Ethos Bioscience)^34^.

### Assessment of kidney function based on creatinine levels

Whole blood was collected from the mice, and the whole blood samples were analyzed immediately on the spot using a portable clinical analyzer, i-STAT Portable Clinical Analyzer (Abbott Laboratories, Abbott Park, IL, USA). A minimum of 100 µl of blood was collected per mouse, and 100 µl of whole blood was loaded into the i-STAT. i-STAT was used to determine the creatinine level in the whole blood.

### Ultrasound examination

Ultrasound examination was performed by using a commercial US scanner (Aplio i800; Canon Medical Systems) with an ultra-high-resolution linear probe (i33LX9, center frequency 33 MHz) and high-resolution linear probe (i18LX5, center frequency 18 MHz), by a board-certificated radiologist (M.S.L. with 11 years of genitourinary imaging experience). After anesthetizing the mouse, the hair around the left flank of the mouse was shaved, the mouse was placed in a prone position, and sterilized ultrasound gel was applied. The location, size and color Doppler imaging of the targeted kidney were performed by using an i33LX9 probe, including measurement of the resistive index (RI). The RI was determined as the mean value of two separate measurements from different interlobar arteries of the targeted kidney. Shear-wave elastography (SWE) was performed by using an i18LX5 under the display setting of 90 kPa. After sufficiently applying gel to the skin of the part to be observed, the probe was closely attached and slowly lifted vertically. When the skin was slightly pulled due to viscosity and the thickness of the ultrasonic gel was approximately 0.5 cm on imaging, the probe was fixed using a clamp. SWE was performed using one-shot mode, which means that the main pulse over one frame was used to measure the resultant speed of the shear wave and elasticity in the tissue being examined. As seen in the figure, two or three 1-mm-diameter linear regions of interest (ROIs) were located based on the information from the propagation map (upper left of Fig. 2D,E) and variation (confidence) map (lower left image of Fig. 2D,E). Per the recommendation of the vender, the radiologist placed the ROI where the contour lines were most parallel and dense on the propagation map, with the lowest variation on the confidence map, and closest to the lower polar cortex on the grayscale map. After four separate shots of the main pulse, 10 to 12 ROIs from four measurements were obtained. Each ROI presents the mean shear wave speed (m/s) in the ROI, as well as the mean stiffness value (kPa) of the ROI calculated from the equation E = 3ρV_s_^2^, where E is the elasticity, ρ is the density of the tissue, and V_s_ is the estimated shear wave speed^35^. The ρ value is regarded as 1 kg/m^3^, as the dominant component of the animal body is water. The median value of the speed (m/s) or stiffness (kPa) is presented as a representative value according to the guidelines proposed by the European Federation of Societies for Ultrasound in Medicine (EFSUMB)^36^.

### Histological analyses

Paraffin-embedded kidney tissue sections (4 μm thick) were stained with Sirius red (all from ScyTek, Logan, Utah, USA) to evaluate the extent of tissue fibrosis. For each kidney section, at least eight fields (at a magnification of × 200) were randomly selected and photographed using a light microscope (BX53F2; Olympus, Tokyo, Japan). The areas of fibrosis and total tissue were measured using ImageJ 1.52d software (Wayne Rasband, National Institutes of Health, USA). A minimum of 10 glomeruli per mouse kidney were evaluated at × 400 magnification. The degree of Sirius red staining of the tubule and glomeruli, as well as the size of the glomeruli, was measured using ImageJ 1.52d software (Wayne Rasband, National Institute of Health, USA)^37^.

Paraffin-embedded pancreatic tissue sections (4 μm thick) were stained with periodic acid Schiff (PAS; ScyTek, Logan, Utah, USA) to evaluate morphologic changes. A minimum of 5 pancreatic islets per mouse were selected and evaluated at × 200 magnification using ImageJ 1.52d software (Wayne Rasband, National Institute of Health, USA).

### Immunohistochemistry

Paraffinized kidney and pancreas tissue blocks were sliced into 4-μm-thick sections, deparaffinized in xylene, and rehydrated in ethanol. The sliced specimens were heated in a microwave oven for 5 min three times with 10% citrate buffer solution (pH 6.0) to retrieve the antigen. Hydrogen peroxide (3%) in methanol was used to block endogenous streptavidin activity for 10 min at room temperature. Sections of the kidney were incubated overnight at 4 °C with primary antibodies against POSTN and TNC. Primary antibodies against insulin and glucagon were incubated overnight at 4 °C with pancreas sections. To detect rabbit and mouse primary antibodies, a Polkin HRP DAB detection kit (GBI Labs, Bothell, WA, USA) was utilized. The sections were counterstained with Mayer’s hematoxylin (ScyTek Laboratories, Logan, UT, USA). The stained slides of kidney tissue were captured in at least eight selected fields and evaluated at × 200 magnification. A minimum of 10 glomeruli per mouse kidney were evaluated at × 400 magnification. The percentage of POSTN- and TNC-positive areas was measured by using ImageJ 1.52d software (Wayne Rasband, National Institutes of Health, USA). At least five pancreatic islets were captured and evaluated at × 200 magnification in the stained slide of the pancreas. The percentages of insulin- and glucagon-positive areas of the pancreatic islets were measured by using ImageJ 1.52d software (Wayne Rasband, National Institute of Health, USA)^38^.

### Immunofluorescence staining

Paraffinized kidney and pancreas tissue blocks were sliced into 4-μm-thick sections, deparaffinized in xylene, and rehydrated in ethanol. The sliced specimens were heated in a microwave oven for 5 min three times with 10% citrate buffer solution (pH 6.0) to retrieve the antigen. Hydrogen peroxide (3%) in methanol was used to block endogenous streptavidin activity for 10 min at room temperature. Sections of the kidney were incubated overnight at 4 °C with primary antibodies against POSTN and TNC. An overnight period was spent attaching secondary antibodies labeled with Cy3 and 488, respectively. We stained the nuclei of the cells with 4’,6-diamidino-2-phenylindole (DAPI, Sigma-Aldrich, St. Louis, MO, U.S.A.). A Leica TCS SP8 STED CW microscope (Leica, Wetzlar, Germany) was used to scan stained slides using confocal microscopy. MetaMorph image analysis software calculated the total intensity per region or field based on the mean signal intensity.

### Establishment of the in vitro model

Cell experiments were conducted in HK-2 cells, a proximal tubule cell line, to confirm the effect of POSTN. A cell line of HK-2 was obtained from the ATCC. During cultivation, the cells were maintained at 37 °C, 95% air, and 5% CO_2_. To prepare complete media for cell culture, 10% fetal bovine serum (FBS; *Biowest,* Nuaillé, *France*) was added to Dulbecco’s modified Eagle’s medium-F12 (Gibco Laboratories, Grand Island, NY, USA). Following one day of starvation, in accordance with the experimental group, the cells were treated with 2 ng/ml TGF-β (R&D Systems, Wiesbaden, Germany) or 0.1 µg/ml, 0.5 µg/ml, or 1 µg/ml rPOSTN (R&D Systems, Wiesbaden, Germany) for 48 h.

This study used INS-1; a rat insulinoma cell line provided by Professor Chul-Woo Yang (Catholic University, Korea). The growing conditions were 37 °C, 95% air, and 5% carbon dioxide. To prepare complete media for cell culture, 10% FBS (Sigma‒Aldrich, St. Louis, MO, USA), 2 mM l-glutamine (Sigma‒Aldrich, St. Louis, MO, USA), 1 mM sodium pyruvate (Sigma‒Aldrich, St. Louis, MO, USA), 10 mM HEPES (Sigma‒Aldrich, St. Louis, MO, USA), and 0.05 mM 2-mercaptoethanol (Sigma‒Aldrich, St. Louis, MO, USA) were added to Dulbecco’s modified Eagle’s medium (Sigma‒Aldrich, St. Louis, MO, USA)^39^. INS-1 cells were treated with STZ (Sigma‒Aldrich, St. Louis, MO, USA) at two concentrations, namely, 2 mM and 4 mM, and anti-POSTN antibodies were added at 0.1 µg/ml, 0.3 µg/ml, and 0.5 µg/ml at each STZ concentration^40,41^. Isotype IgG (Abcam, Cambridge, MA, USA) was treated at 0.5 µg/ml as an anti-POSTN antibody-treated control (R&D Systems, Wiesbaden, Germany). The viability of the cells was measured using EZ-Cytox (DoGenBio, Seoul, Korea) following the above experiment^39,42^.

### Western blot analysis

A diabetic mouse model was maintained for 24 weeks, and the kidneys and pancreas were collected at the end of the experiment. Both whole kidney tissues and pancreas tissues from mouse and HK-2 were homogenized. The proteins were isolated from tissues and cells using RIPA buffer (Thermo Fisher, Rockford, IL, USA.). Protein concentrations in each sample were determined by the BCA assay (Thermo Scientific, Rockford, IL, USA), and western blotting was conducted using the same protein concentrations. Using sodium dodecyl sulfate/polyacrylamide gel electrophoresis (SDS/PAGE), the same amount of sample was separated based on protein size. To probe target proteins, separated proteins were transferred and immobilized onto membranes (Millipore Corporation, Bedford, MA, USA). Following the blocking of nonspecific proteins, the membranes were incubated with specific primary antibodies overnight at 4 °C. In order to preserve the antibody and protein, the membrane was cut, and then the primary antibody was attached. A membrane was fragmented based on the kda of each protein, and the membrane fragments were then attached overnight to each primary antibody. Anti-rabbit IgG or anti-mouse IgG antibodies (all from Cell Signaling Technology, Danvers, MA, USA) were used as secondary antibodies. On a chemiluminescence system (Advansta, CA, USA), protein bands were visualized and quantified using ImageJ 1.52d software (Wayne Rasband, National Institute of Health, USA).

### Statistical analysis

The data presented in this manuscript are expressed as the means with standard deviations. In the bar graphs, the mean values are presented along with standard deviations. The statistical analysis was conducted using IBM SPSS 20.0 and GraphPad Prism 8.0 (GraphPad Software, San Diego, CA). DN patients were analyzed using one-way ANOVA to evaluate the differences between groups. The Mann‒Whitney test was used to compare groups, including the WT STZ vs. KO STZ groups. One-way ANOVA was applied to analyze cell culture-related experimental results. *P* < 0.05 was considered statistically significant.

### Supplementary Information

Supplementary Tables.

Supplementary Figures.

## Data Availability

The datasets generated and/or analyzed as a part of the current study are available from the corresponding author on reasonable request.

## Abbreviations

DN: Diabetic nephropathy
POSTN: Periostin
rPOSTN: Recombinant periostin
TNC: Tenascin C
STZ: Streptozotocin
*Postn*-null: Periostin knockout B6 mice
UNXSTZ: Unilateral nephrectomy–STZ injection model
Cr: Creatinine
