# Supplementary material for: Periostin deficiency attenuates kidney fibrosis in diabetic nephropathy by improving pancreatic β-cell dysfunction and reducing kidney EMT
Source: Sci Rep. 2023 Oct 16;13:17599. doi: 10.1038/s41598-023-44177-5 (PMC10579313; doi:10.1038/s41598-023-44177-5)
Supplement: Supplementary file 1 — Supplementary Tables. [file 41598_2023_44177_MOESM1_ESM.pdf]

Supplementary Table S1. The sources of antibodies used in western blot

**Antibodies**	**Company**	**Catalogue number**	**Location**
		**primary antibody**	
anti-β-actin	Sigma-Aldrich	A1978	St. Louis, MO
anti–α-SMA	Abcam	ab5694	Cambridge, MA
anti-TNC	Abcam	ab108930	Cambridge, MA
anti-col1a1	Lsbio	LS-C343921	Seattle, WA
anti-TGFβ1	Abcam	ab92486	Cambridge, MA
anti-psmad23	Abclonal	AP0548	Woburn, MA
anti-smad23	cell signaling technology	#8685	Danvers, MA
anti-smurf1	Santa Cruz Biotechnology	sc-100616	Santa Cruz, CA
anti-GAPDH	cell signaling technology	#2118	Danvers, MA
anti-p38	cell signaling technology	4511	Danvers, MA
anti-p-p38	Abclonal	A14401	Woburn, MA

Supplementary Table S2. The sources of antibodies used in IHC

Antibodies	Company	Catalogue number	Location
anti-periostin	Abcam	ab14041	Cambridge, MA
anti-TNC	Abcam	ab108930	Cambridge, MA
anti-insulin	Santa Cruz Biotechnology	Sc-8033	Santa Cruz, CA
anti-glucagon	Abcam	ab10988	Cambridge, MA

Supplementary Table S3. The sources of antibodies used in IHC

Antibodies	Company	Catalogue number	Location
anti-tenascin C	Abcam	ab108930	Cambridge, MA
anti-periostin	R&D system	AF2955	Boston, MA
cy3 conjugated affinipureF (ab)fragment	Jackson human reaserch	705-166-147	West Grove, PA
alexa fluor 488	Invitorgen	a21206	Carlsbad, Ca
