# Supplementary material for: Periostin deficiency attenuates kidney fibrosis in diabetic nephropathy by improving pancreatic β-cell dysfunction and reducing kidney EMT
Source: Sci Rep. 2023 Oct 16;13:17599. doi: 10.1038/s41598-023-44177-5 (PMC10579313; doi:10.1038/s41598-023-44177-5)

What we used

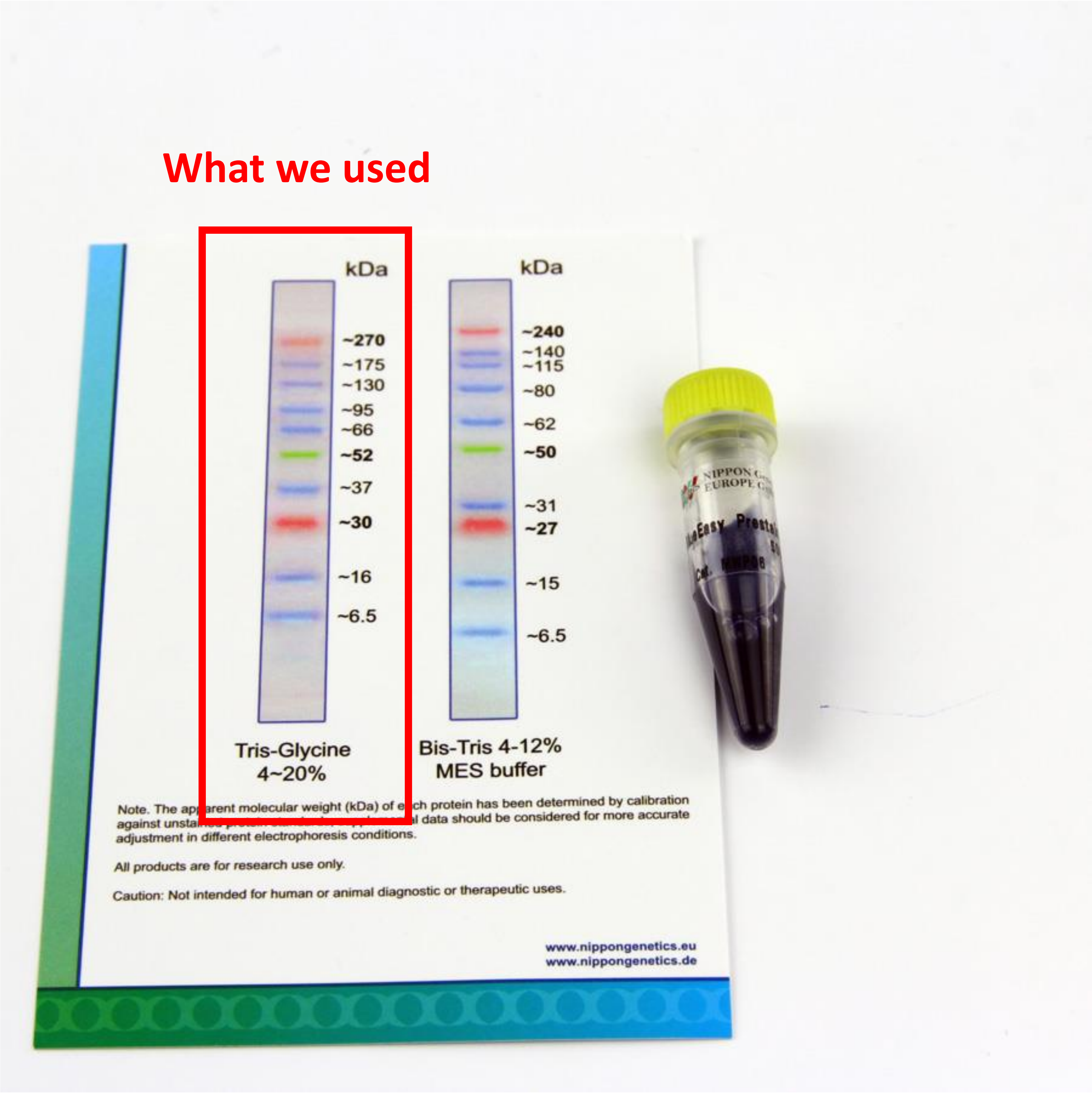

kDa
~270
~175
~130
~95
~66
~52
~37
~30
~16
~6.5
Tris-Glycine 4~20%
kDa
~240
~140
~115
~80
~62
~50
~31
~27
~15
~6.5
Bis-Tris 4-12% MES buffer
adjustment in different electrophoresis conditions.
All products are for research use only.
Caution: Not intended for human or animal diagnostic or therapeutic uses.
www.nippongenetics.eu
www.nippongenetics.de

Figure 1a
(band section)

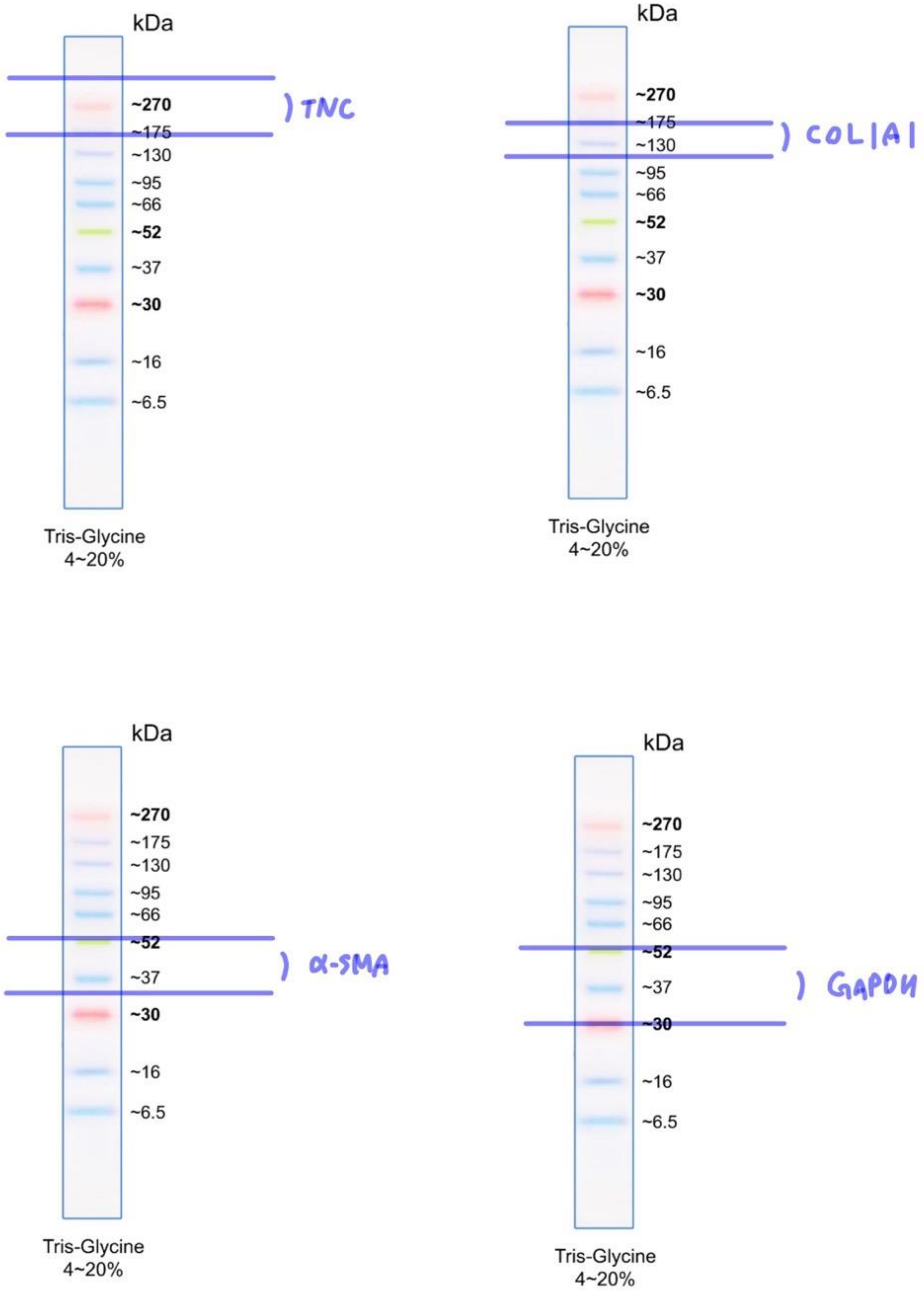

Figure 1b

Tenascin C

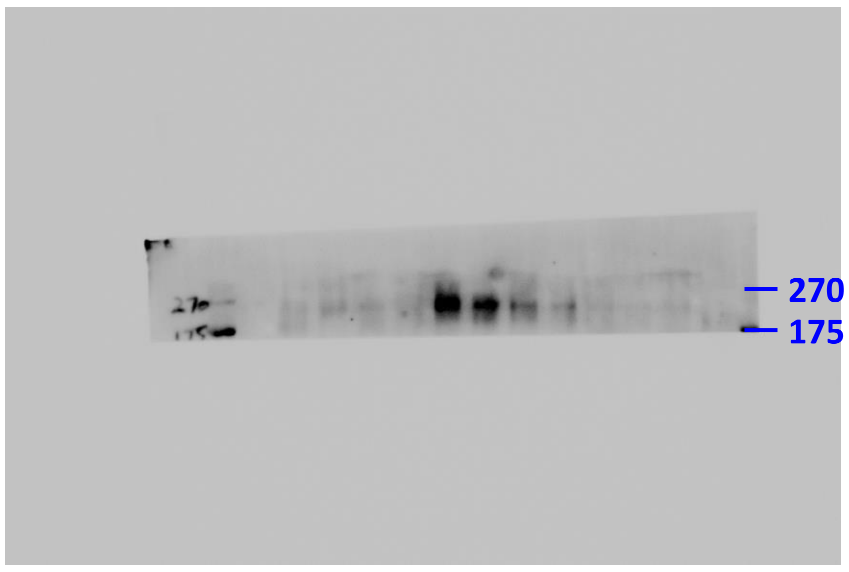

COL1A1

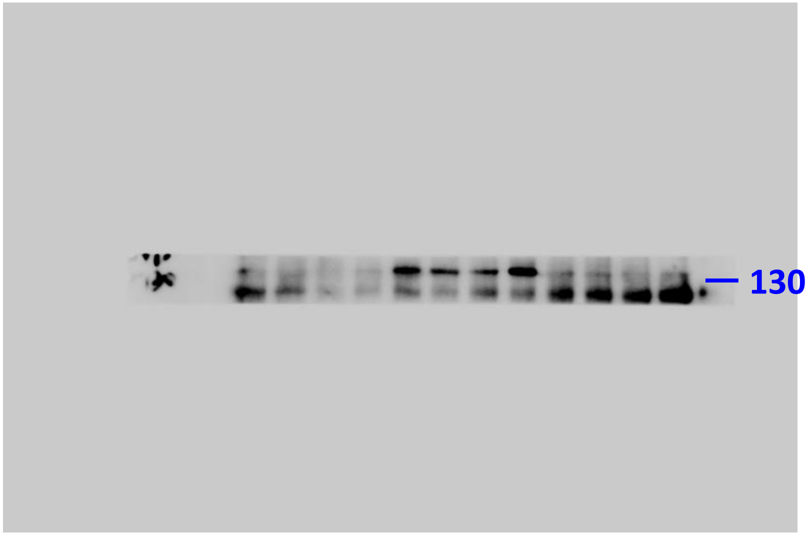

α-SMA

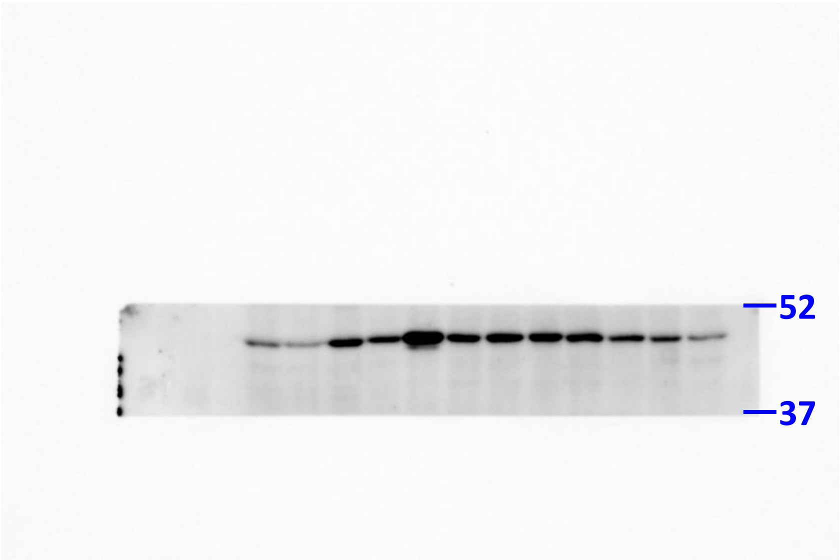

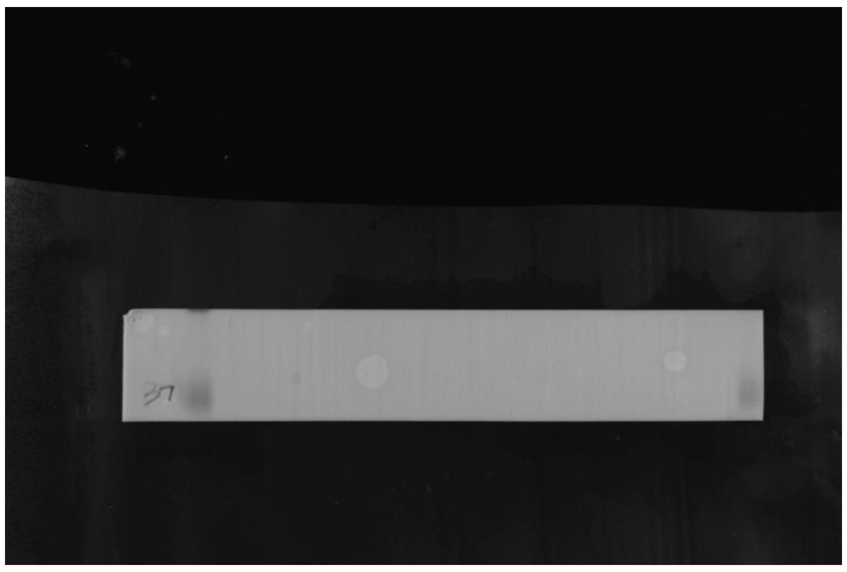

GAPDH

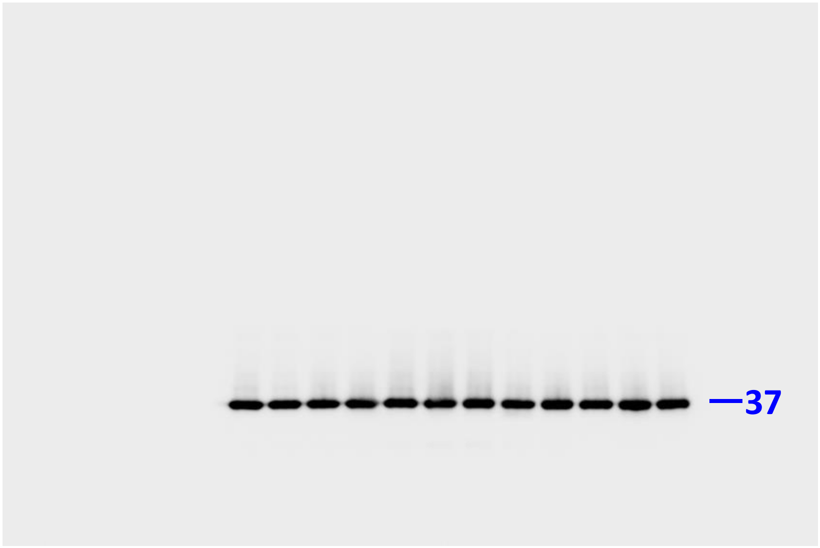

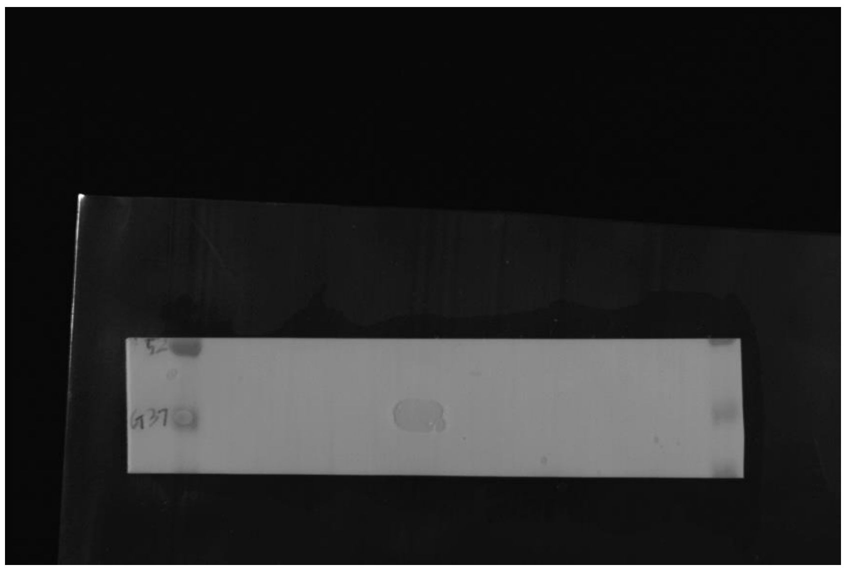

Figure 2a
(band section)

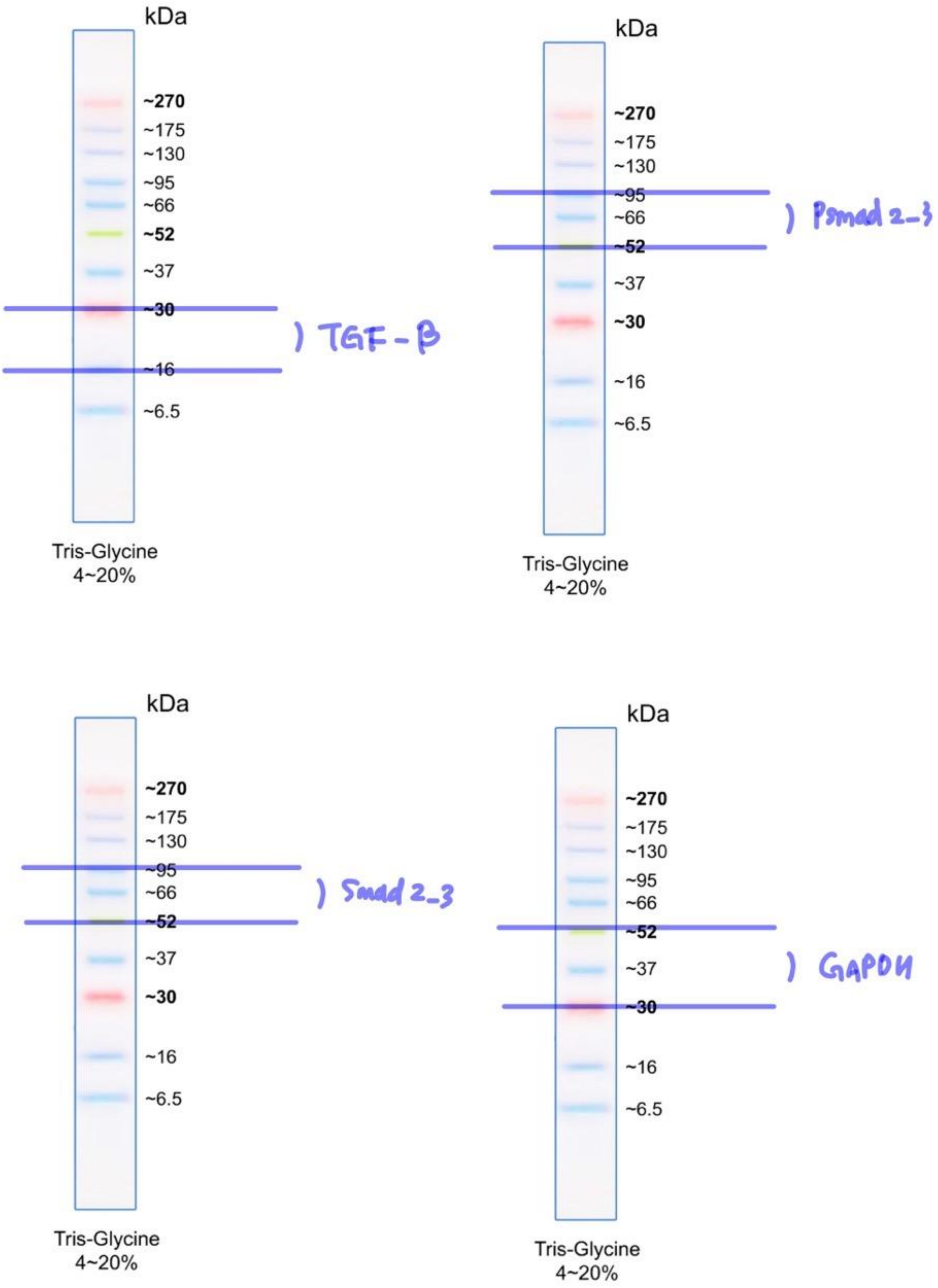

Figure 2b

TGFb

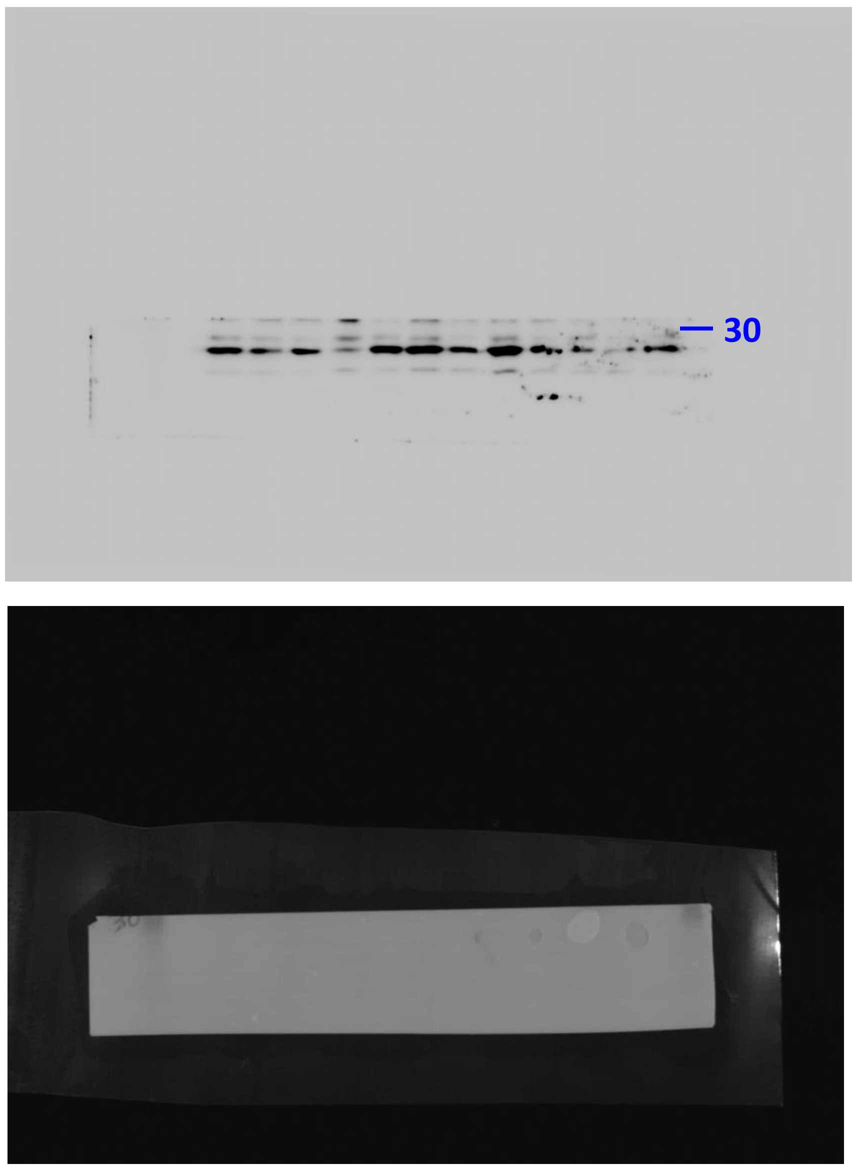

Psmad 2/3

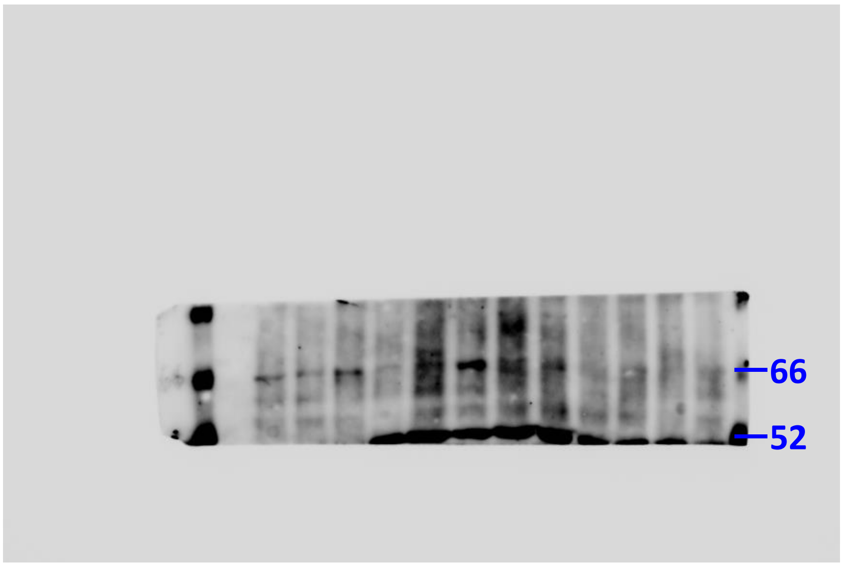

ssmad 2/3

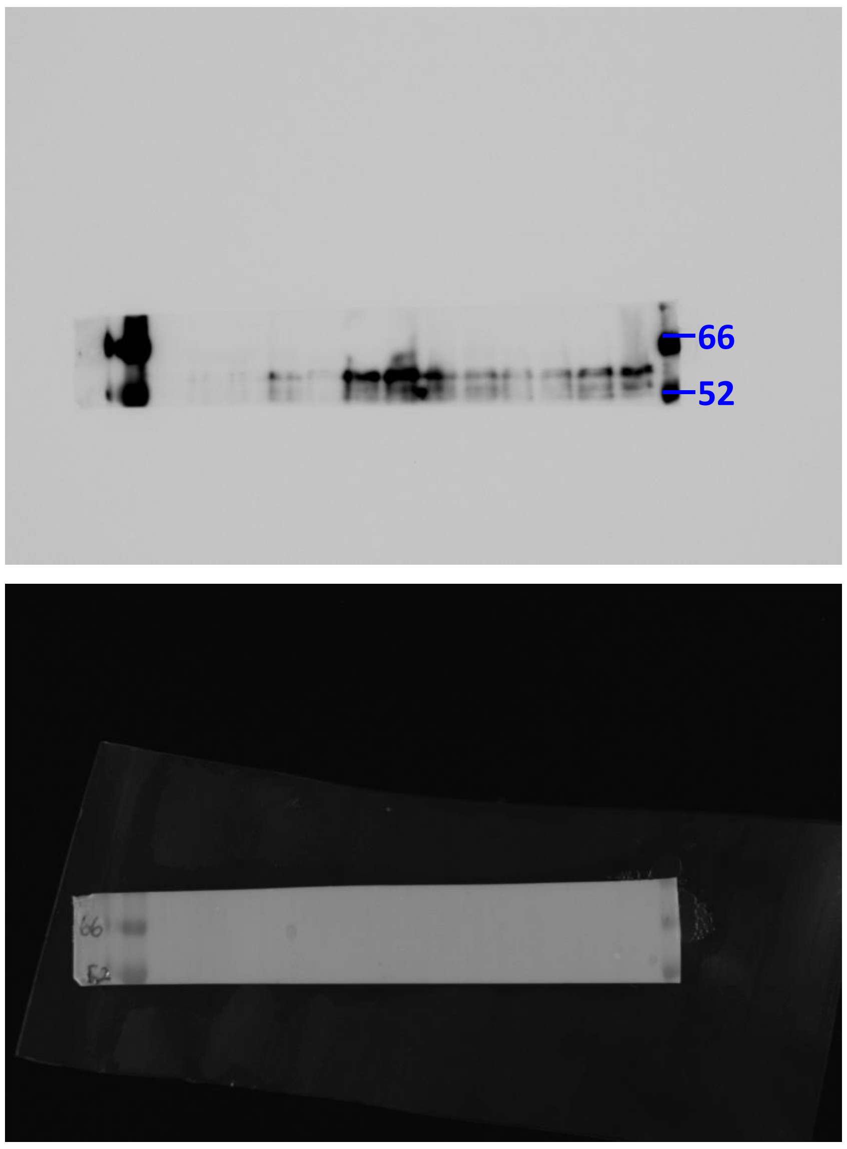

GAPDH

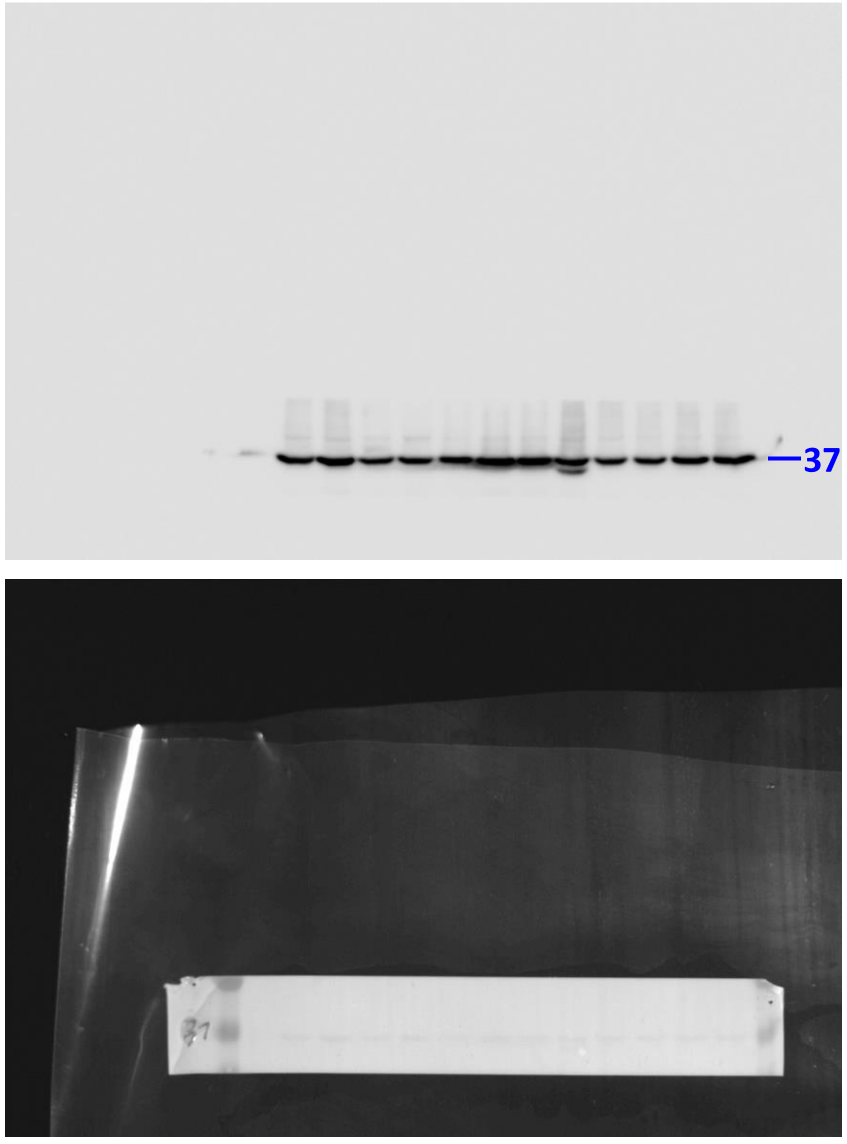

Figure 3a
(band section)

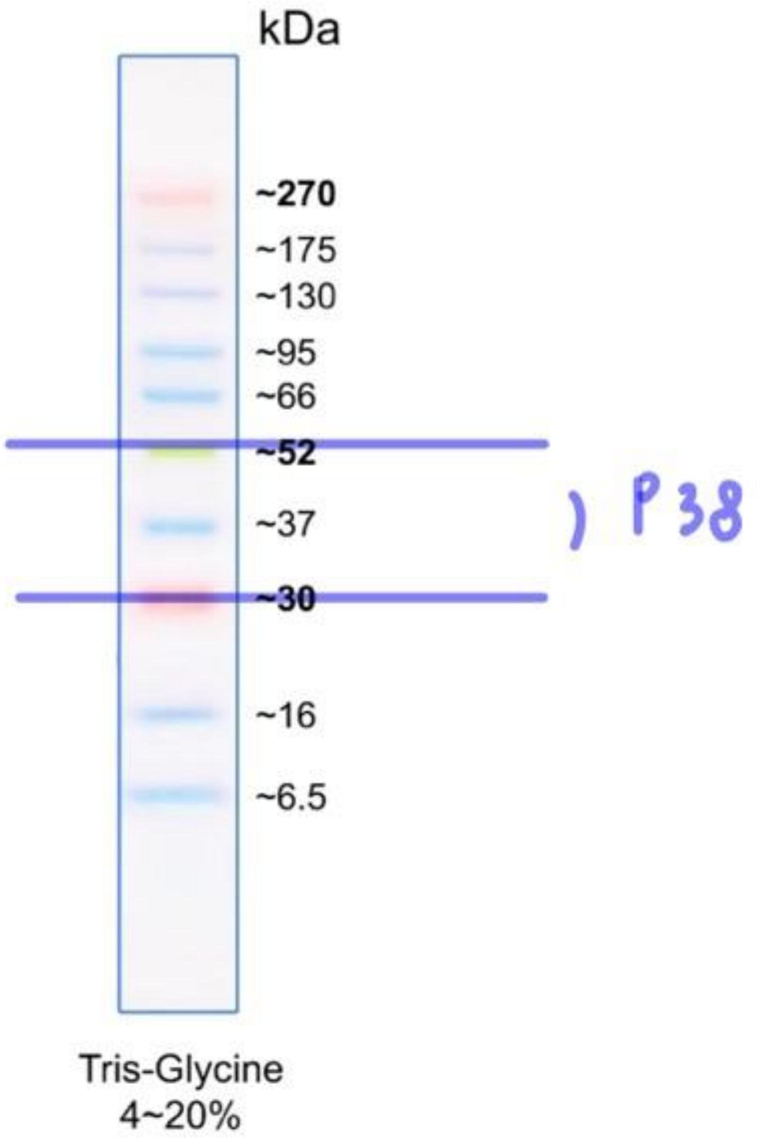

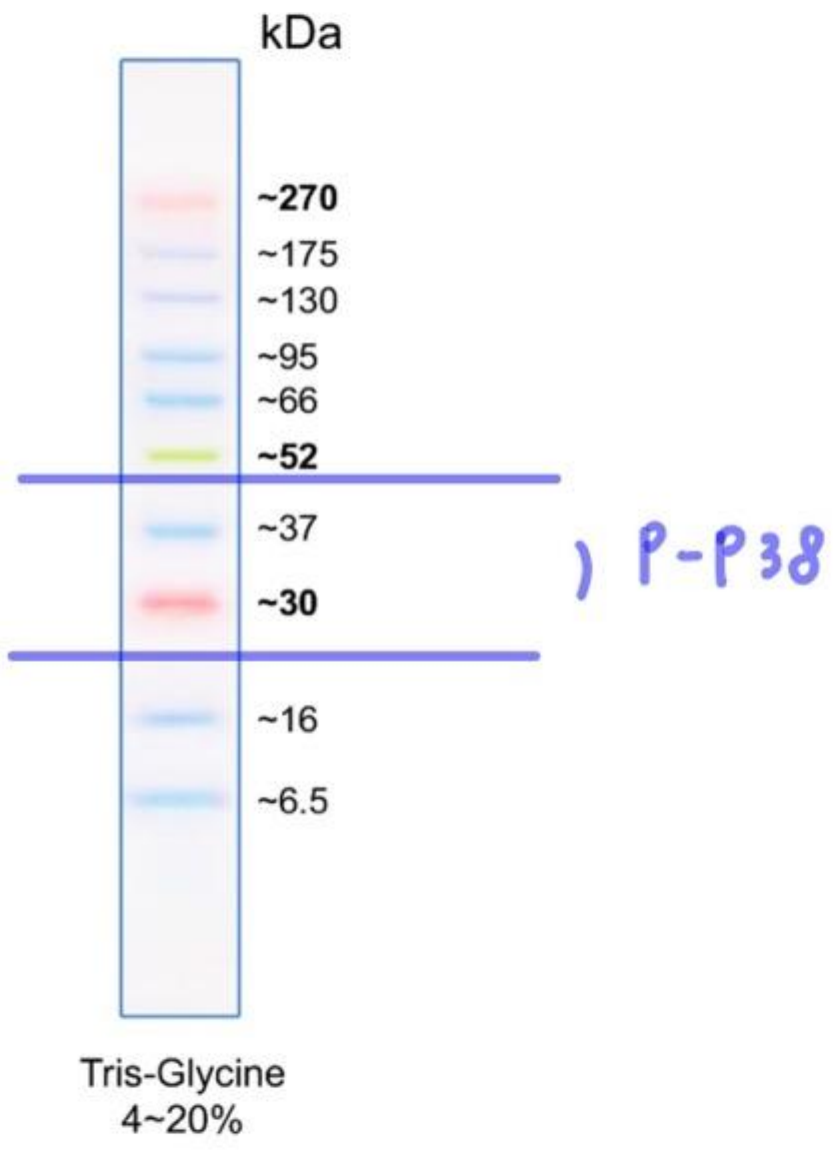

Figure 3b

p38

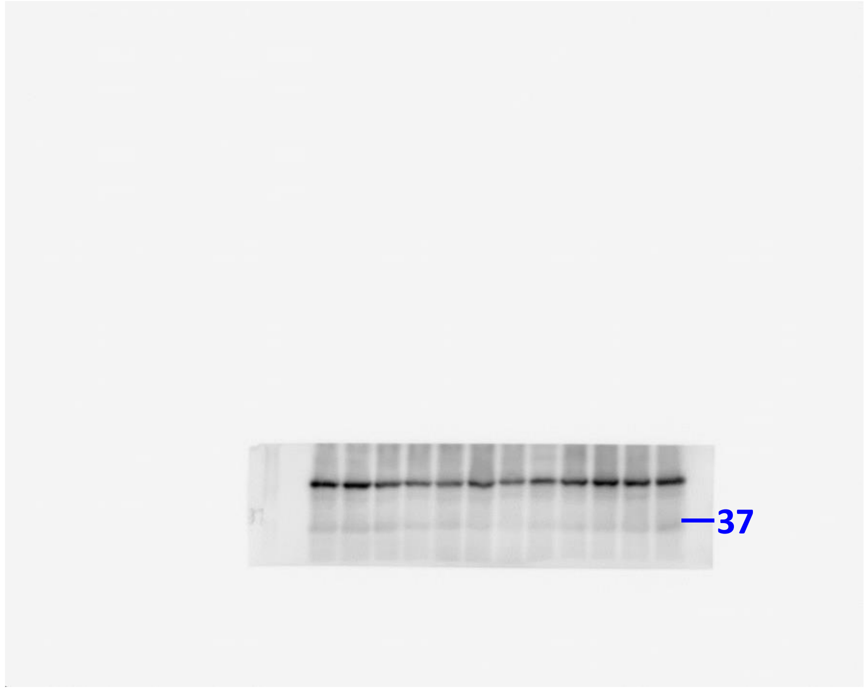

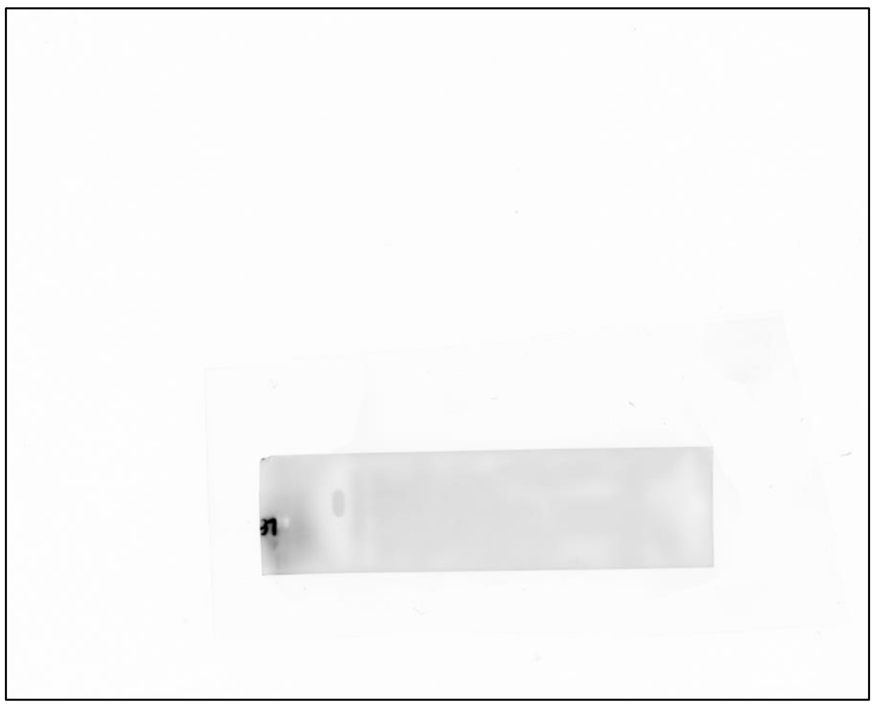

pp38

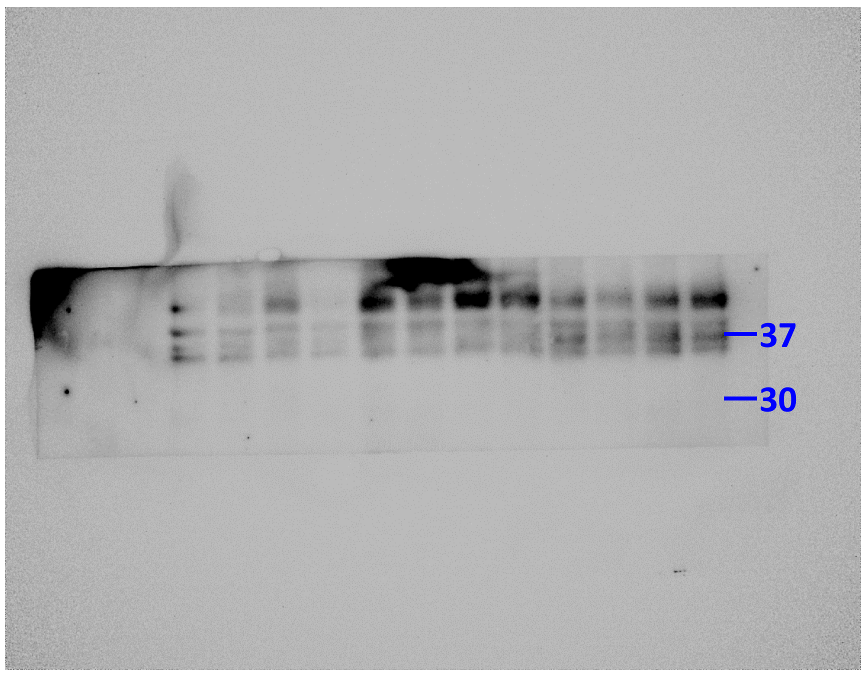

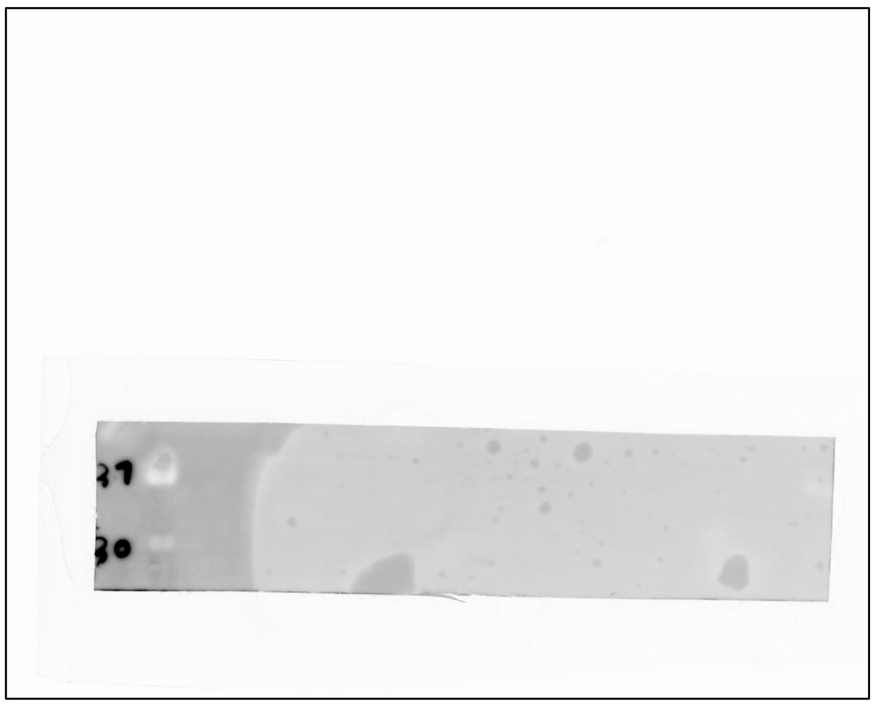

Figure 4a
(band section)

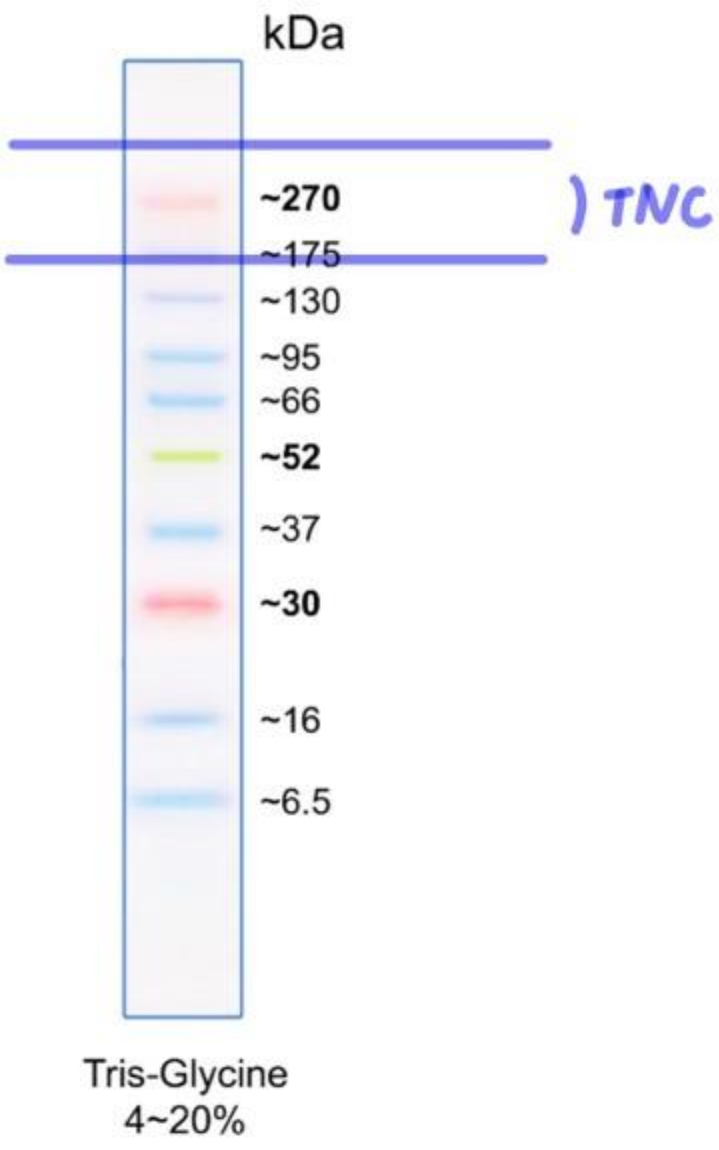

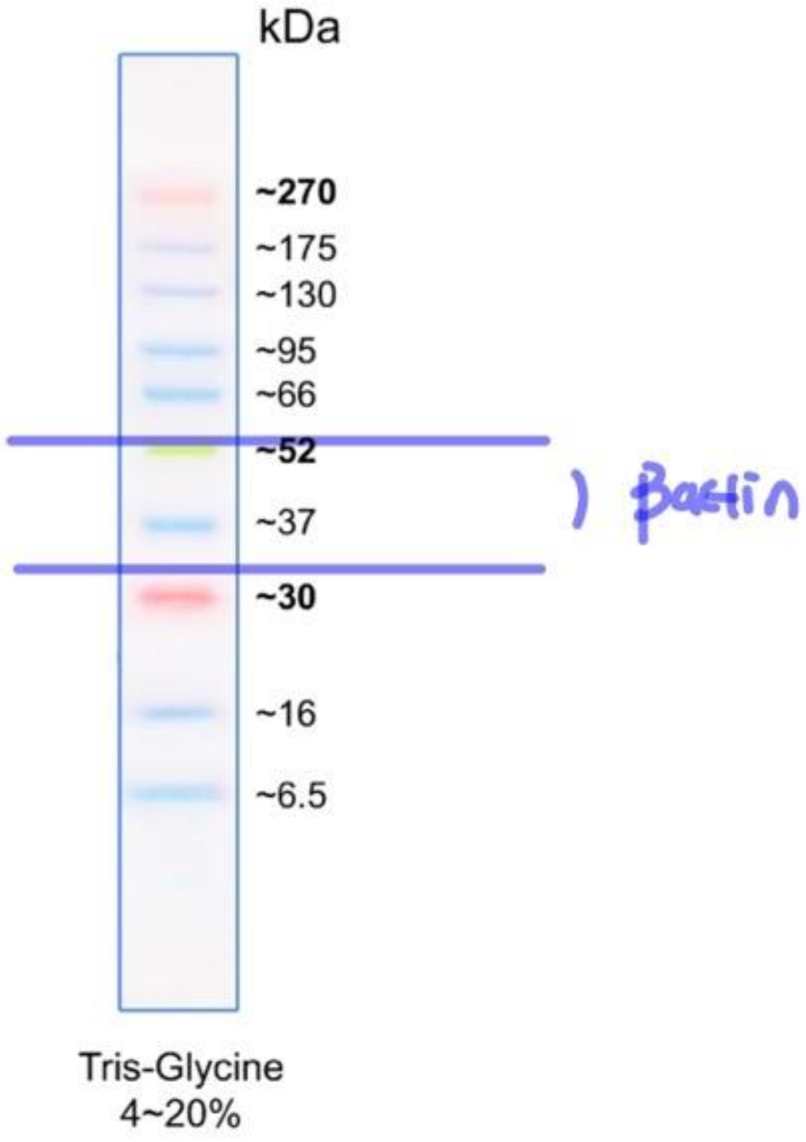

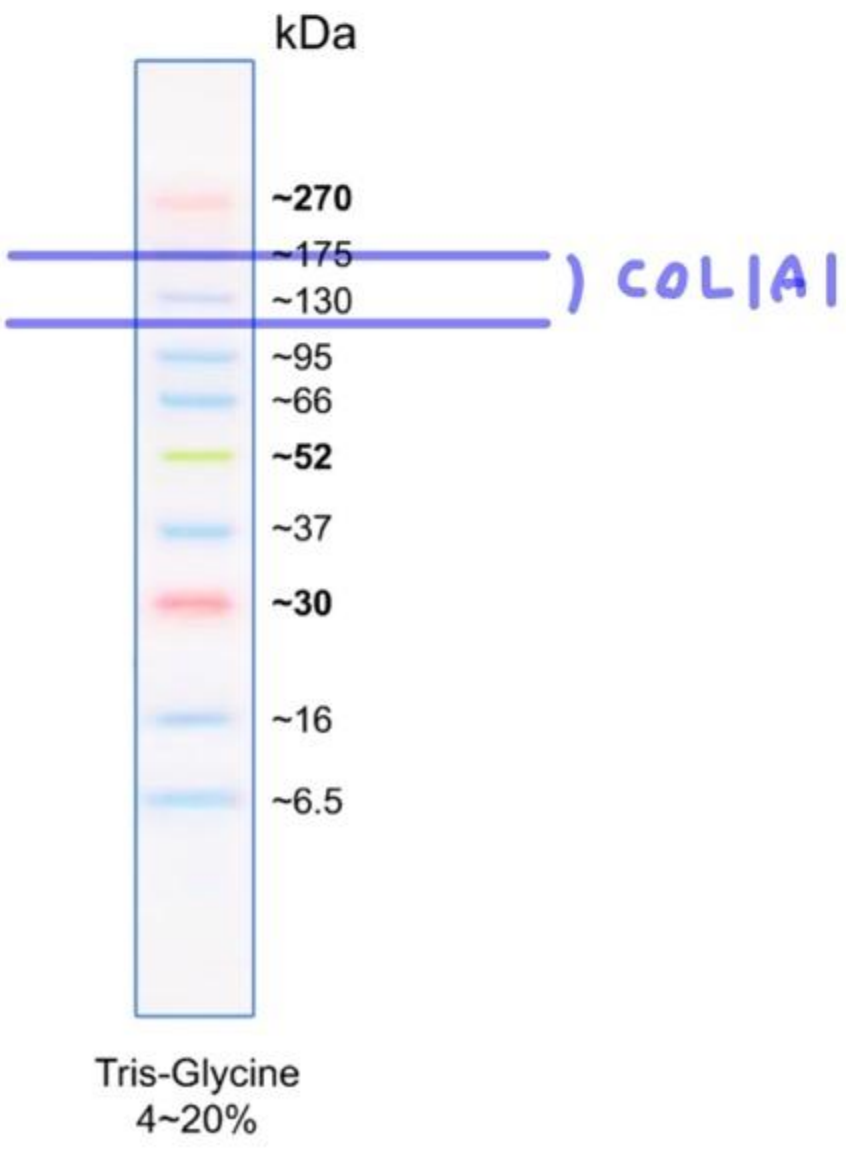

Figure 4b

TNC

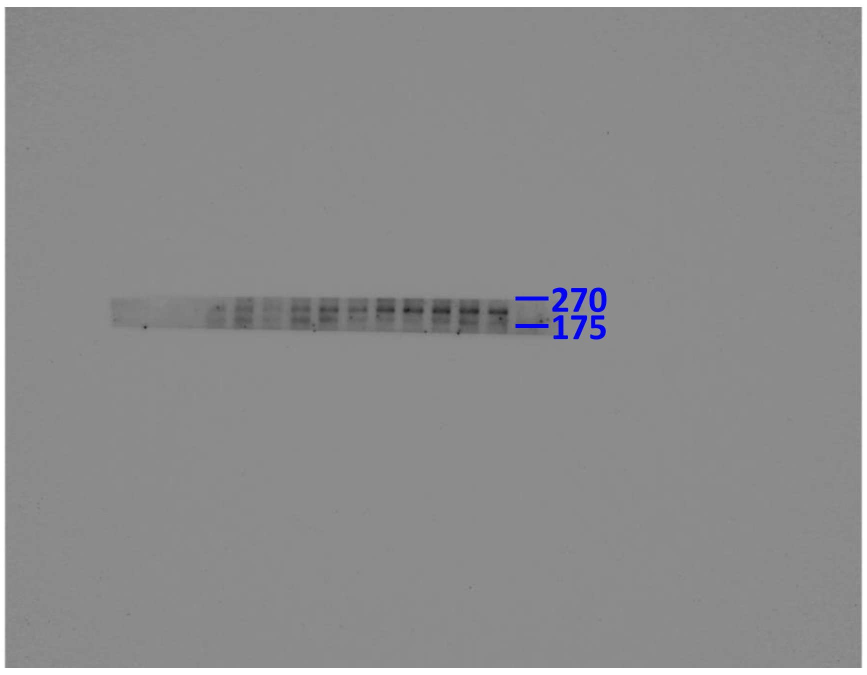

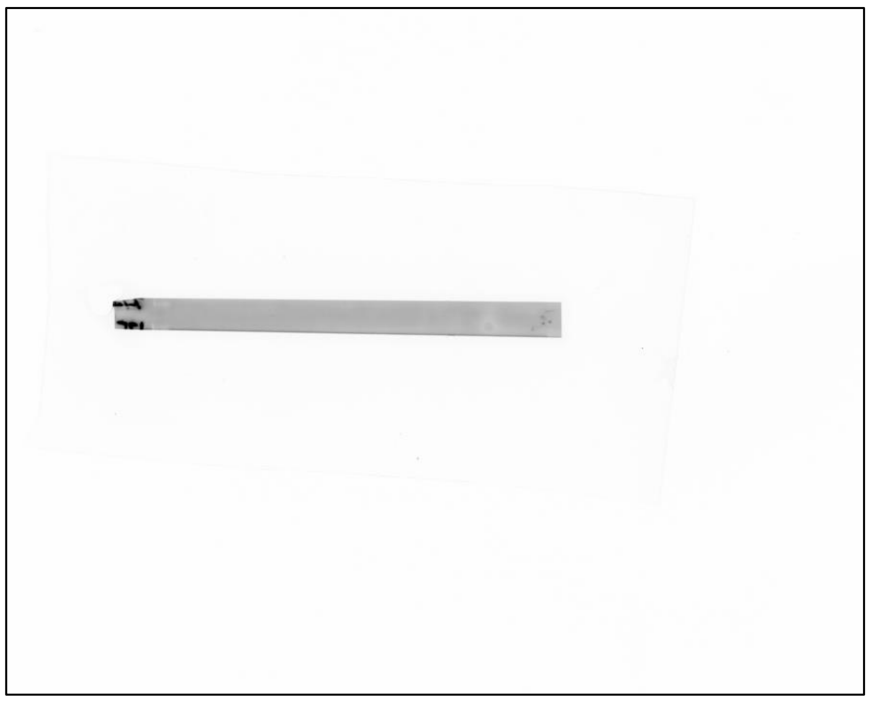

COL1A1

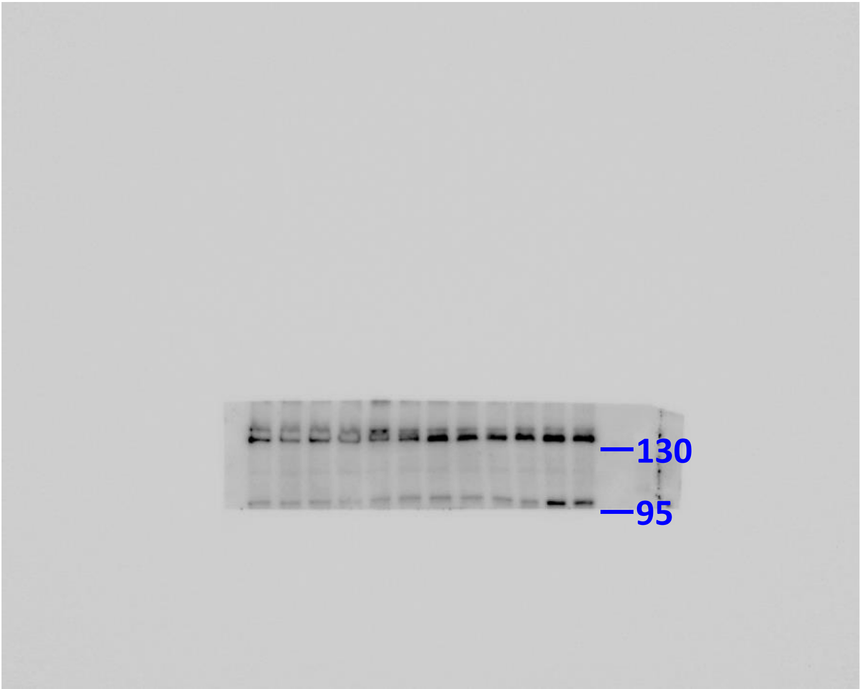

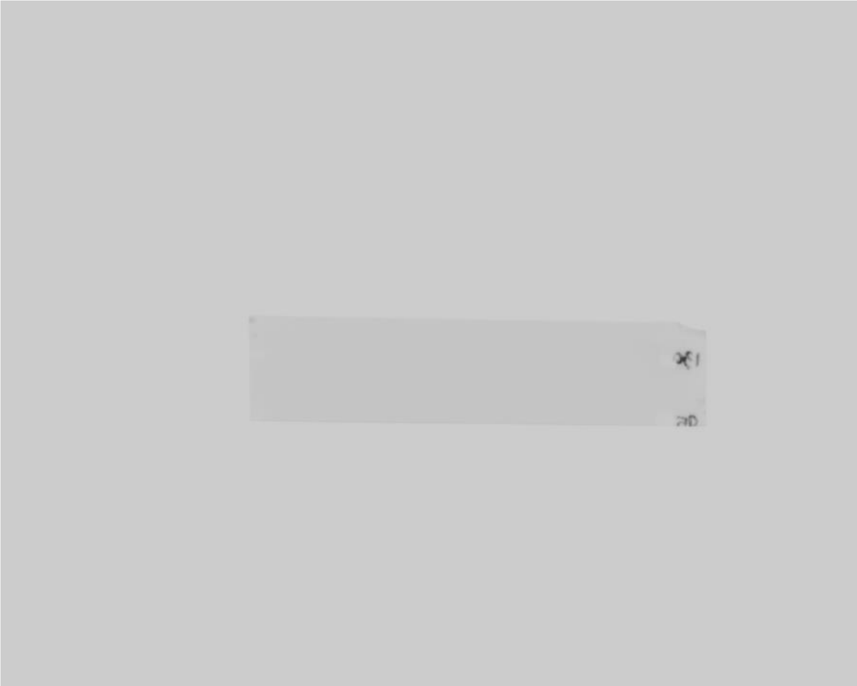

B-actin

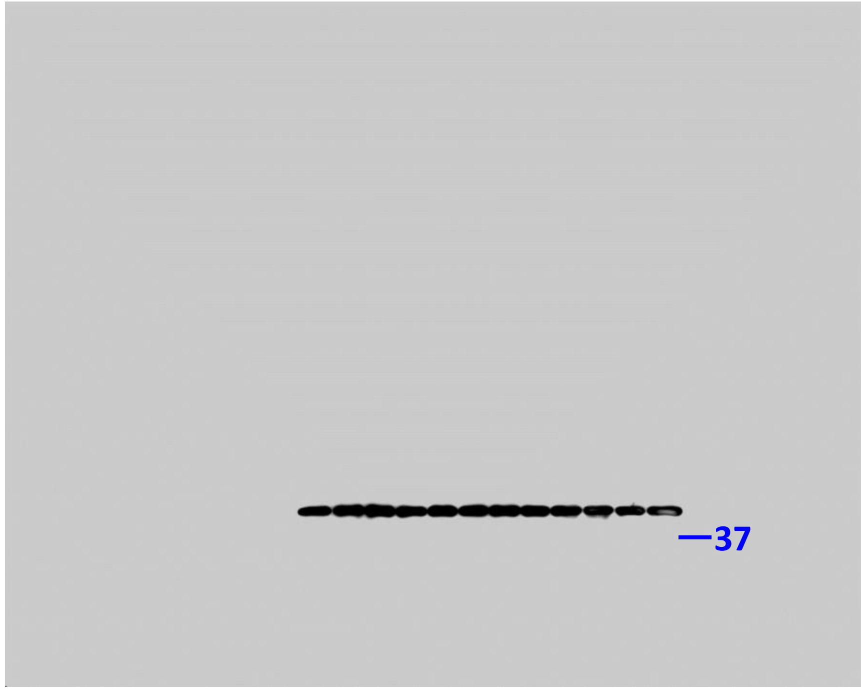

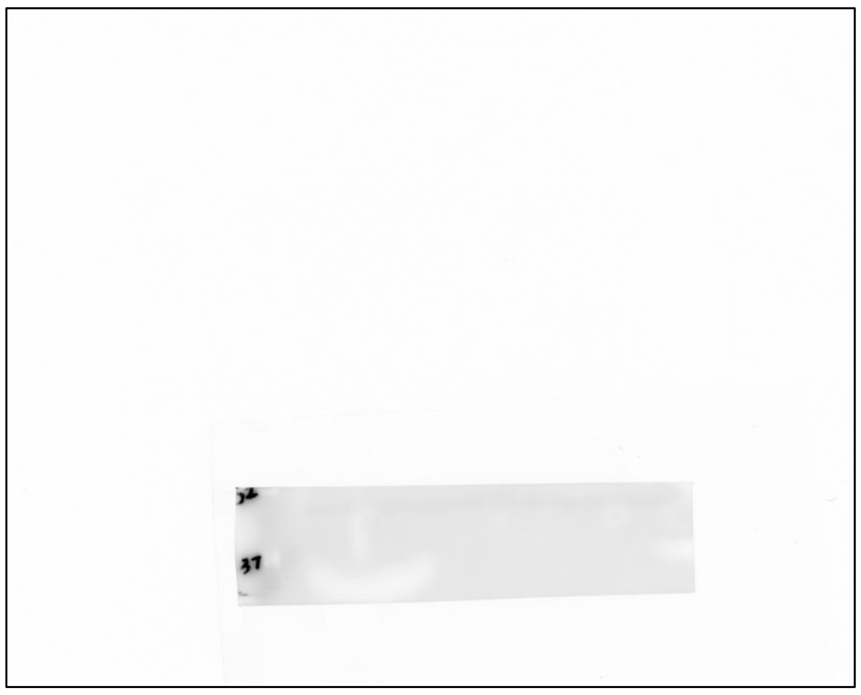

Figure 5a
(band section)

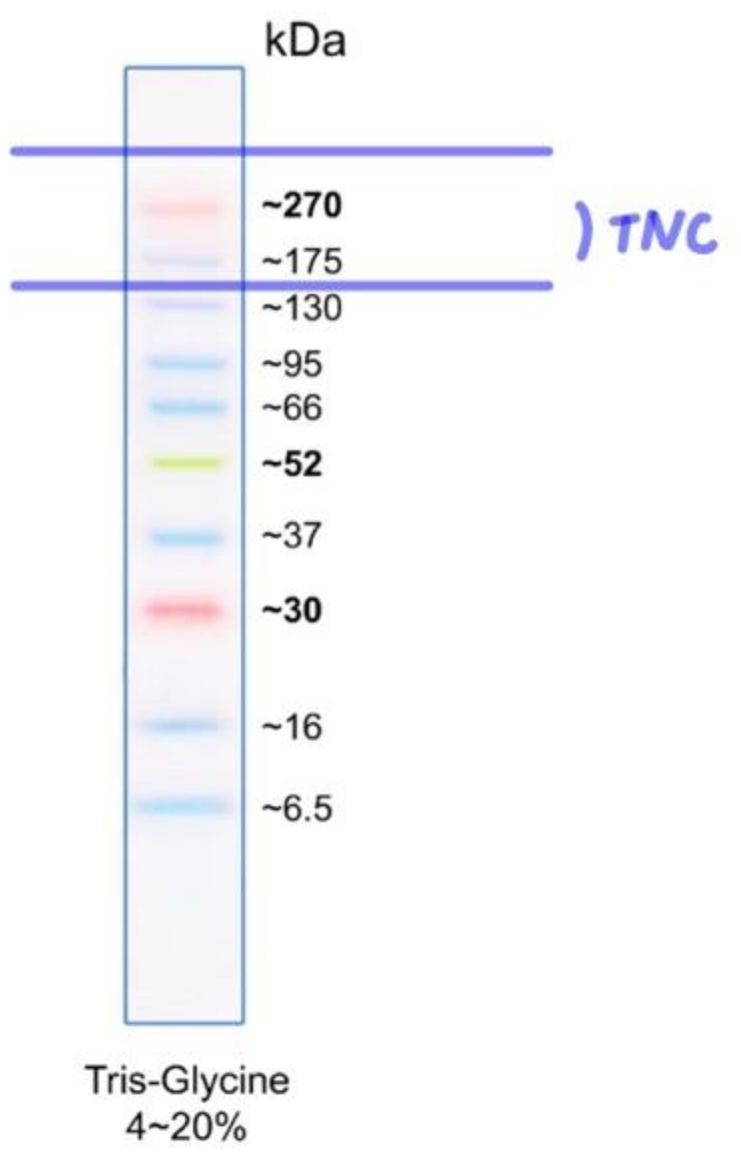

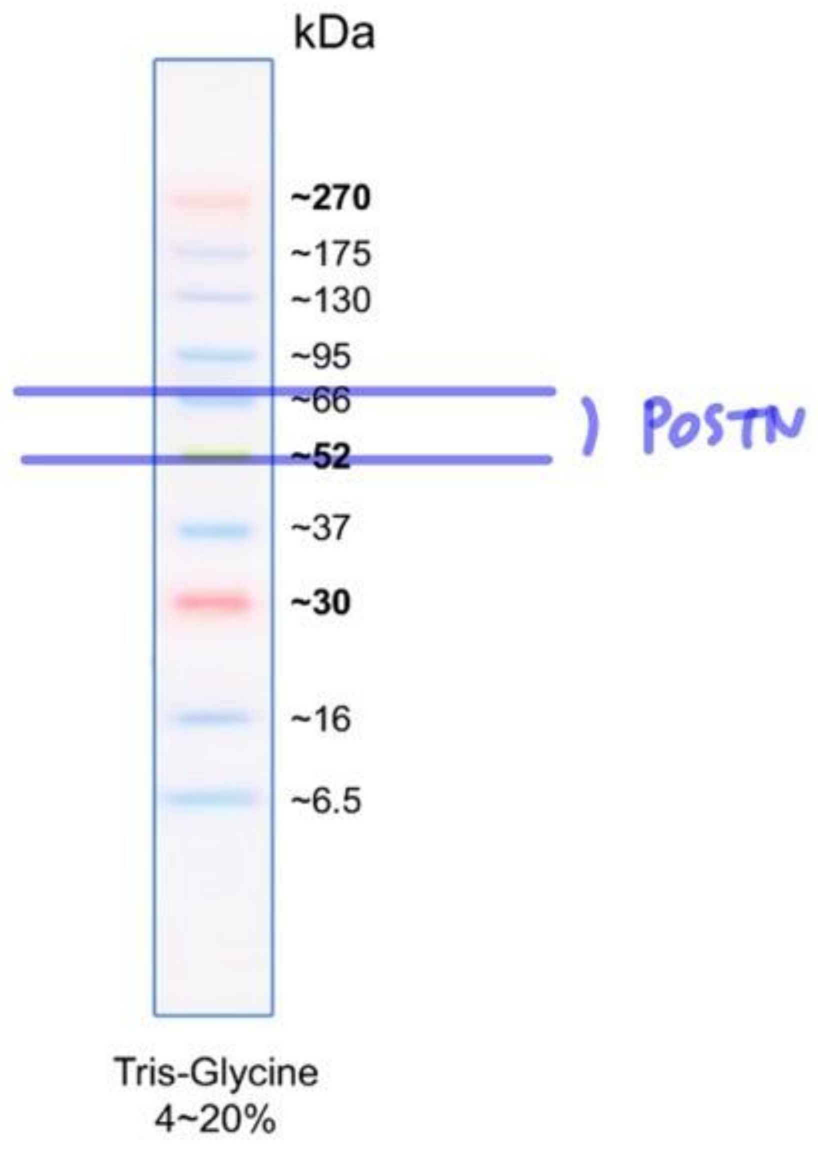

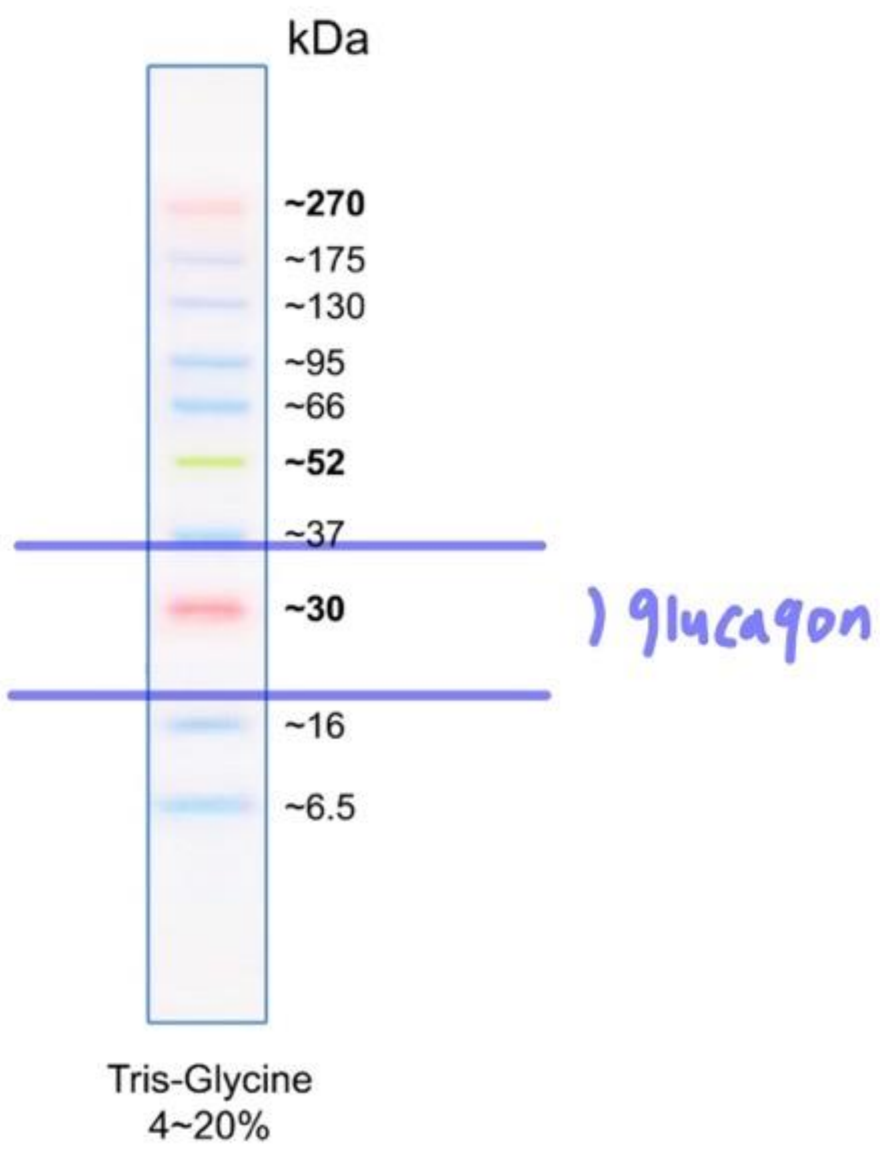

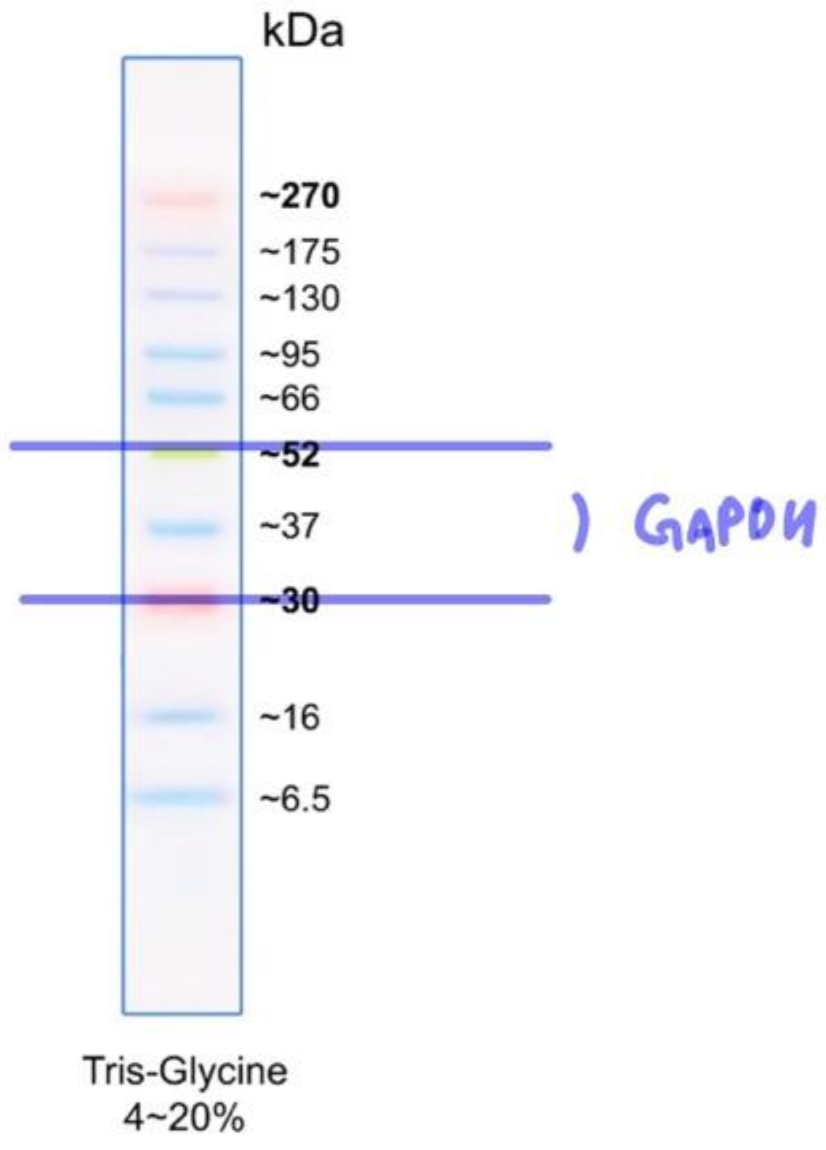

Figure 5b

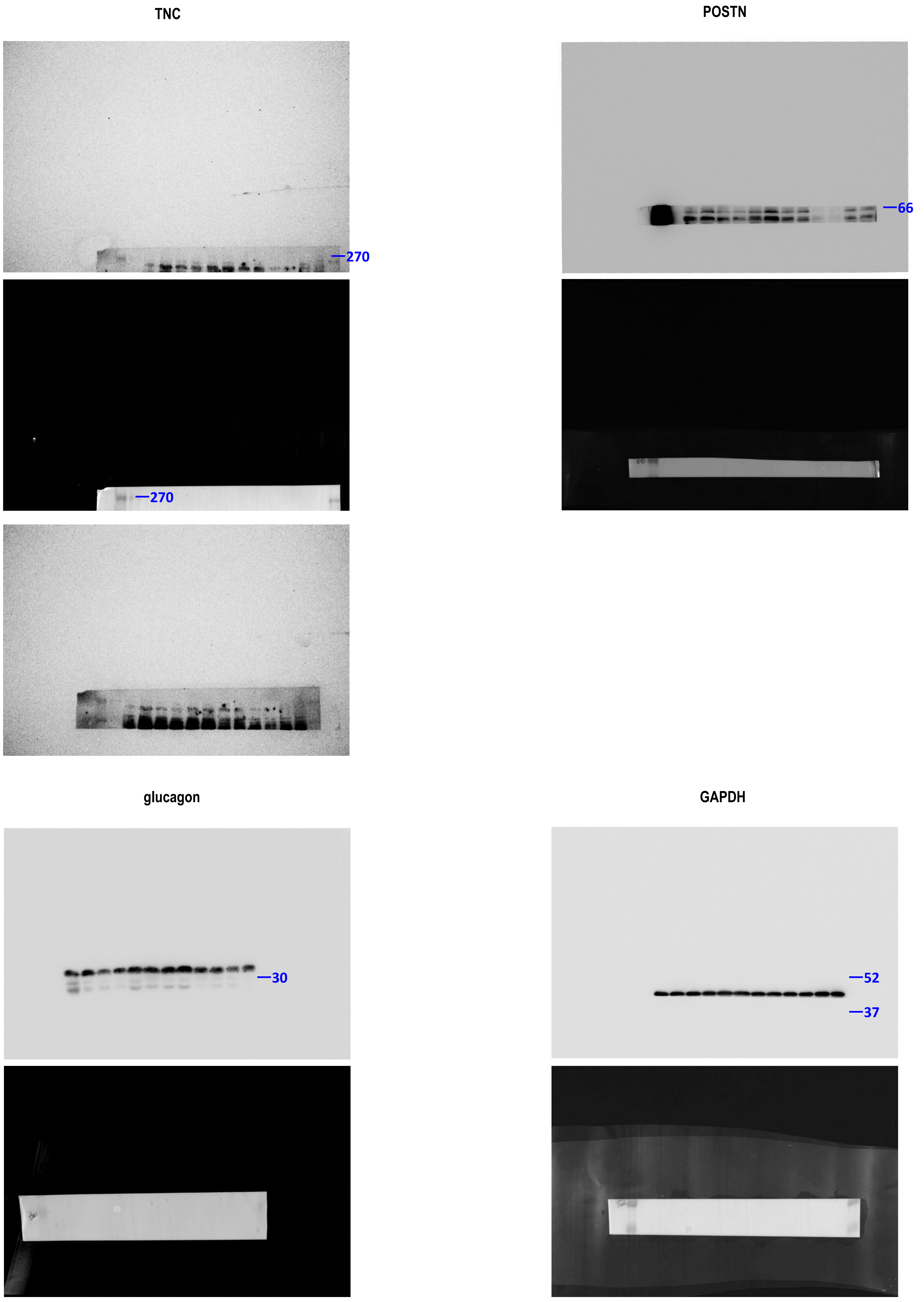

Supplement: Supplementary file 2 — Supplementary Figures. [file 41598_2023_44177_MOESM2_ESM.pdf]
